# Contraction criteria for incremental stability of differential systems with discontinuous right-hand sides^☆^

**DOI:** 10.1016/j.heliyon.2022.e12621

**Published:** 2022-12-22

**Authors:** Yingying Lang, Wenlian Lu

**Affiliations:** a School of Mathematical Sciences, Fudan University, Shanghai 200433, PR China; b Shanghai Center for Mathematical Sciences, Fudan University, 200433, PR China; c Shanghai Key Laboratory for Contemporary Applied Mathematics, 200433, PR China

**Keywords:** Incremental stability, Filippov systems, Exponential convergence, Discontinuous right-hand sides

## Abstract

Incremental stability analysis, which plays a crucial role in dynamical systems, especially nonlinear systems, has attracted more and more concern for its applications in real world control systems nowadays. This paper presents a constructive approach to derive sufficient conditions for incremental exponential stability of the Filippov solutions of a class of differential systems with discontinuous right-hand sides, by introducing a sequence of continuous dynamical systems which is uniformly contracting and approximating the Filippov systems in terms of the evolution map graphs. Afterwards, several applications of the derived theoretical results are explored. Some specific classes of control dynamical systems with discontinuous right-hand sides are studied and relative detailed conditions are presented to show the power of the present approach to investigate the stability of switched dynamical systems, Hopfield neural network with discontinuous activations and sliding mode control.

## 1 Introduction

Stability analysis researches the long-term behavior of systems' steady states, which nowadays plays a more and more important role in complex systems science. In 2004, Incremental stability [1], [2] has been presented as an excellent instrument for stability analysis, which is able to address problems of synchronization of coupled systems.

A dynamic system is said to be incrementally stable if its trajectories with different initial states converge to each other as time goes to infinity. Incremental stability problems attract more and more concern in recent years for the potential applications in some frontier fields, such as PI controlled missile [3] and some problems about synchronization of dynamical networks [4], [5], [6]. Several literatures [7], [8] discussed the problems along these lines, and some examples of the applications are included in [9].

Then people encounter the problem of testing incremental stability properties. It is proved that the method based on Lyapunov function and contraction metrics, which is often used in stability problems, also helps deal with the study of incremental stability in [10].

Contraction theory, which is a key theory used in this paper, is also a classical tool widely used to obtain the criteria of dynamic systems for incremental stability [11], [12], [13], [14]. A dynamic system is called contracting if its trajectories approach to a steady-state solution regardless of its initial states. In general, if the right-hand side of the dynamical system is continuously differentiable, the problem of incremental stability proof is much easier, because the corresponding Jacobian matrix exists and thus the vector field can be simply constructed. For dynamical systems with discontinuous right-hand sides, some techniques need to be exploited to obtain the criteria for contraction.

There are several classical theories which are also shown to be applicable to systems with discontinuous right-hand sides. Several conditions for the existence and uniqueness of Caratheodory solutions, which should be guaranteed before contraction analysis, are discussed and concluded in [15].

Exponential stability, which is a special case of stability problems and has attracted a lot of interests for its wide applications in complex systems science, including some specific networks, e.g., memristor-based recurrent neural networks [16], which plays a vital part in associative memory and optimistic computation. In these specific applications, we need to guarantee exponential stability for the Filippov systems.

In recent few years, there has been growing concerns on stability analysis of Filippov systems and a number of related works and findings arising. Under the circumstance that the value function associated to the minimization problem is locally Lipschitz continuous, sufficient conditions for local stability in the sense of Filippov solutions were given in [17]. The concept of the Filippov solution was exploited to study the dynamics of a class of delayed dynamical systems with discontinuous right-hand sides [18]. Several criteria were derived to ensure the global asymptotic stability of the error system for the delayed neural networks with discontinuous activation functions under the framework of Filippov solution [19], and detailed criteria were proposed to guarantee the exponential stability of switched Filippov systems using approximation [20].

This paper uses approximation method to research the criteria for contraction property of Filippov solutions of a class of dynamical systems with discontinuous right-hand sides. The related criteria, which is proved sufficient for exponential stable systems, is proposed here. Several primary conditions are listed and need to be satisfied in order to guarantee local existence and uniqueness of their Filippov solution. Under the conditions mentioned above, by approximating the system with a sequence of continuously differential systems, a series of conditions are proposed so that the Filippov system is exponentially stable, that is, its solutions consequently converge to one another exponentially. Through this method, sufficient conditions are proposed for exponential convergence of the Filippov systems. And then, with the theorem we proved, several applications to specific models are given. Corresponding detailed conditions for the systems' exponential stability under several specific situations are listed and several numerical examples are shown to support our conclusions afterwards.

The paper is organized as follows. In Section 2, our model formulation and the assumptions for the existence and uniqueness of the Filippov solution of the dynamical systems with discontinuous right-hand sides are presented. In Section 3, we will introduce the contraction theory, on the basis of which we proposed our main theorem, researching the conditions for incremental stability of the differential systems with discontinuous right-hand sides. In Section 4, several typical applications of the main theory are presented, and relative conclusions on several kinds of systems are provided to demonstrate our main theorem. Illustrative numerical examples are shown to belabor the effectiveness of our results in Section 5.

## 2 Preliminary

This section focuses on the model formulation of Filippov systems with respect to the dynamical systems with discontinuous right-hand sides. A special class of the systems, the switched system, is also modeled for specific analysis afterwards. Before our analysis, we need to guarantee that the Filippov solutions of the Cauchy problem of (2) have a unique solution. So several assumptions are proposed here.

### 2.1 Model formulation

A Filippov system can be formulated as follows.

(1) $$\overset{˙}{x}=f(x,t)$$

where $x\in R^{n}$, the function $f:R^{n}\times [0,+\infty )\to R^{n}$ is discontinuous w.r.t. *x* and *t*. The solution of the system (1) can be defined as a solution of the following differential inclusion (Page 101-103 in [21]), with the initial state $x(0)=x^{0}$:

(2) $$\overset{˙}{x}\in K[f](x,t),$$

in which

$$K[f](x,t)=\underset{\epsilon >0}{⋂}\underset{\mu (P)=0}{⋂}\bar{co}\{f(B(x,\epsilon )\setminus P,t)\},$$

where μ(⋅) represents the Lebesgue measure, B(x,ϵ)={y:|y−x|≤ϵ} represents the *ϵ*-neighborhood of *x* with the given vector norm |⋅|, and $\bar{co}$ represents convex closure. For some $Z\subset R^{n}$, let $\left|Z|=\sup_{x\in Z}|x\right|$.

Then we consider the switched system as follows, which is a special class of dynamical systems with discontinuous right-hand sides, as given in Sec. 2 of [22]. For more details, the readers are referred to Chapter 2 of [15] and Page 101-103 in [21] directly.

(3) $$f(x,t)=f_{i}(x,t),\;(x,t)\in R_{i}$$

with $f_{i}:R^{n}\times R^{+}\to R^{n}$ and regions $R_{i}\subset R^{n}\times R^{+}$ for i=1,⋯,K. All $R_{i}$ have nonempty interior. The discontinuities of (1) compose of several smooth hypersurfaces of dimension m(m<n). Suppose that ${\left\{S_{i}\right\}}_{i=1}^{N}$ is a sequence of smooth hypersurfaces of dimension (n−1), $S_{i}=\{(x,t)\in R^{n}\times R^{+}:\phi_{i}(x,t)=0\}$, where $\phi_{i}(x,t)\in C^{1}(R^{n}\times R^{+})$, and the continuous regions of *f* is a sequence of connected regions divided by the hypersurfaces $S_{i}(i=1,2,...,N)$. The switching hypersurfaces may intersect and generate a manifold of lower dimensions. Let $E_{i}^{+}(E_{i}^{-})$ be a connected region satisfying the following conditions:

1. $\partial E_{i}^{+}(\partial E_{i}^{-})\subset {⋃}_{k}S_{k}$;
2. f(x,t) is continuous on $E_{i}^{+}(E_{i}^{-})$;
3. $\phi_{i}(x,t)<0(>0)$ holds on $E_{i}^{-}$ ($E_{i}^{+}$).

With the notations above, for each *i*, $E_{i}^{+}$ and $E_{i}^{-}$ are two different regions with their common boundary on $S_{i}$.

### 2.2 Existence and uniqueness conditions

Before our main result, we need to first ensure existence and uniqueness of the Cauchy problem of Filippov system (2). Herein, we present several existing results on this issue. Readers are referred to [15], [22], [23] for the details. First, let F(x,t)=K[f](x,t). ‘Upper semicontinuity’ for the set-valued map *F* is defined as follows.

Definition 1 **Sec. 1, Chap. 2 in [23]**

A set-valued map $F:R^{n}\times R^{+}⇉Y$ is called upper semicontinuous at $(x,t)\in R^{n}\times R^{+}$ if and only if for any neighborhood U of F(x,t), ∃δ>0, such that $\forall (\overset{˜}{x},\overset{˜}{t})\in B((x,t),\delta )$, $F(\overset{˜}{x},\overset{˜}{t})\subset U$.

From Definition 4 and 5 in [22], the following assumption should be firstly satisfied so that the existence of the solution of (2) is guaranteed:

Assumption 1 **[22]**

The set-valued map $F:R^{n}\times R^{+}⇉R^{n}$ satisfies that for all $(x,t)\in R^{n}\times R^{+}$, F(x,t) is non-empty, bounded, convex and closed, and *F* is upper semicontinuous at (x,t). |F(x,t)|≤a(t)|x|+b(t) for all $(x,t)\in R^{n}\times R^{+}$ with bounded functions a(t) and b(t) on $R^{+}$, |F(x,t)−F(y,t)|≤h(|x−y|) for some continuous function $h(\cdot ):R^{+}\to R^{+}$.

Thus, if Assumption 1 is satisfied, then system (2) has at least one solution that can be extended to $R^{+}$ for any initial value at $t_{0}=0$ (Chapter 2 in [15]).

Furthermore, under the conditions in Assumption 1, the following assumption guarantees that the Filippov solution of (2) is unique.

Assumption 2 **Sec. 3.8, Chap. 2 in [24]**

There are some $C^{1}$ function $V:R^{n}\times R^{+}\to R^{+}$ with V(0,t)=0 and V(z,t)>0 for all z≠0 and t≥0, and some measurable function $\phi :R^{n}\times R^{n}\times R^{n}\times R^{n}\times R^{+}\to R^{+}$ such that

$${\left(\frac{\partial V}{\partial z}\right)}^{⊤}(x-y,t)(p-q)+\frac{\partial V}{\partial t}(x-y,t)\leq \phi (x,y,p,q,t)V(x-y,t)$$

for all $x,y\in R^{n}$, p,q∈F(x,t), and all t≥0.

For the switched system formulated as (3), an alternative sufficient condition was presented by Theorem 2, Sec. 10, Chapt. 2 in [15] to guarantee the uniqueness of the Filippov solution.

Take system (3), K=2 and N=1, as an example. As defined in (3), for a multidimensional case in domain *E*, with its corresponding connected regions $E^{+}$ and $E^{-}$ divided by switching hypersurface *S*, let $f^{+}(x,t)$ and $f^{-}(x,t)$ be the limiting values of the function f(x,t) at the point (x,t), x∈S, from the regions $E^{+}$ and $E^{-}$ respectively. $f_{N}^{+}(x,t)$ and $f_{N}^{-}(x,t)$ are the projections of the vectors $f^{+}(x,t)$ and $f^{-}(x,t)$ onto the normal vector to *S* directed from $E^{+}$ to $E^{-}$ at the point (x,t). With the notations defined above, we have the following Assumption 3 and Lemma 2, Lemma 3, which are important theorems proposed in [15].

Assumption 3 **Theorem 2, Sec. 10, Chapt. 2 in [15]**

With the notations above, for each t∈(0,+∞) at each point x∈S, at least one of the inequalities $f_{N}^{-}(x,t)>0$ or $f_{N}^{+}(x,t)<0$ (possibly different inequalities for different *x* and *t*) is fulfilled.

From Theorem 2, Sec. 10, Chapt. 2 in [15], under Assumption 3, right uniqueness of Filippov solution for switched system (3) (K=2, N=1) occurs for t∈(0,+∞) in the domain *E*.

In conclusion, we have lemmas for existence and uniqueness of Filippov solution as follows,

Lemma 1 **[15], [24]**

*With*Assumption 1, Assumption 2*, the existence and uniqueness of Filippov solution for system*(2)*occurs for*t∈(0,+∞)*in*$R^{n}$*.*

Lemma 2 *From Theorem 2, Sec. 10, Chapt. 2 in**[15]**, under*Assumption 3*, right uniqueness of Filippov solution for the bimodal case of switched system*(3)*(*K=2*,*N=1*) occurs for*t∈(0,+∞)*in the domain E.*

For more general switched systems defined as (3), we need to prove the uniqueness of the solution with concrete analysis. The following lemma may help.

Lemma 3 **Theorem 1 in Sec. 10, Chapter 2 in [15]**

*Let a function*f(t,x)*in a domain D is continuous only on a set M of measure zero. Let there exist a summable function*l(t)*such that for almost all points*(t,x)*and*(t,y)*of the domain D we have*|f(t,x)|≤l(t)*and for*$|x-y|<\epsilon_{0}$*,*$\epsilon_{0}>0$*,*

$$(x-y)\cdot (f(t,x)-f(t,y))\leq l(t)|x-y{|}^{2}$$

*Then under the simplest convex definition (Page 50 in**[15]**), the equation*$\overset{˙}{x}=f(t,x)$*has right uniqueness in the domain D.*

We take a simple model with two switching surfaces intersecting each other for example and research the conditions for uniqueness in Appendix A for illustration.

## 3 Contraction analysis for the Filippov system

In this section, our main theorem is expounded, together with the detailed proof. Several basic definitions for incremental exponential stability and relative primary contraction theory are introduced first, which is the foundation for the main theorem.

### 3.1 Incremental stability and contraction theory

Here we present some primary definitions and introduce the contraction properties and relative theory for differential systems. Before contraction analysis, firstly, we here introduce the definition of matrix measure, denoted by the function $\nu (\cdot ):R^{n\times n}\to R$.

Definition 2 **Definition 1 in [20]**

For any real matrix $X\in R^{n\times n}$ and a given norm |⋅|, we define the corresponding matrix measure ν(X) as

$$\nu (X)=\lim_{h\to 0^{+}}\frac{|I+hX|-1}{h}.$$

The matrix measure above can be thought of as the one-sided directional derivative of the induced matrix norm function |⋅|, evaluated at the point *I*, in the direction of *X*.

Consider the ordinary differential system as follows, which is generally time-dependent:

(4) $$\overset{˙}{x}=f(x,t).$$

Consider the differential equation (4) and two of its solutions $x(t)=\phi (t,t_{0},x_{0})$ and $y(t)=\phi (t,t_{0},y_{0})$ with initial time $t_{0}$ and initial value $x_{0}$ and $y_{0}$ respectively. We have the following definition of incremental exponential stability (IES):

Definition 3 **Definition 5 in [20], detail in [12]**

Let $C\subset R^{n}$ be a forward invariant set for (4) and |⋅| be some norm on $R^{n}$. The system (4) is called incrementally exponentially stable (IES) in C if there exist constants λ>0 and M≥1 such that

$$|x(t)-y(t)|\leq Me^{-\lambda (t-t_{0})}|x_{0}-y_{0}|,\forall t\geq t_{0},\forall x_{0},y_{0}\in C.$$

Remark 1 The definition IES here is independent of the initial time $t_{0}$, i.e., the constants *K* and *λ* are independent of $t_{0}$.

Remark 2 There are some other types of stability property, for instance, incrementally asymptotical stability [20], asymptotical periodic stability [25], etc. The property IES here implies that the solutions of the system will converge towards each other at an exponential speed with regard to the difference of their initial values, which is stricter than incrementally asymptotical stability. Under the circumstance that the system is incrementally exponentially stable and has a periodic solution, the system's solutions will converge to the periodic trajectory at an exponential speed, called asymptotical periodic solutions (seen in Example 4 in [20] and system (22) in [18]). If the system has a solution converging to an equilibrium, the property ‘incremental exponential stability’ implies that the dynamics with other initial values will converge to the equilibrium as well (seen in Example 1 and 2 in Section 5 in this paper). If the system has a solution converging to the discontinuous surface, e.g. system (25), with the property ‘incremental exponential stability’, the dynamics with different initial values will also converge to the discontinuous surface, which helps to deal with sliding mode control problems (seen in Section 4.5 and Example 3 in Section 5).

A supplementary definition of K-reachable Sets is presented as follows.

Definition 4 **Sec. 4.4 in [26]**

Let K>0 be any positive real number. A subset $C\subset R^{n}$ is K-reachable if for any two points $x_{0}$ and $y_{0}$ in C, there is some continuously differentiable curve γ:[0,1]→C such that $\gamma (0)=x_{0}$, $\gamma (1)=y_{0}$, and $\left|\gamma^{\prime }(r)|\leq K|y_{0}-x_{0}\right|$ for all r∈[0,1].

We then introduce the concept of infinitesimal contraction and the contraction theory [13] for systems with continuously differentiable right-hand sides. Denote the whole state place of system (4) by Σ, $\Sigma \subseteq R^{n}$.

Definition 5 **Sec. 1.1 in [13]**

The continuously differentiable vector field defined by f(x,t) in (4) is said to be (infinitesimally) contracting in a *K*-reachable set C⊂Σ if there exists some norm in C, with associated matrix measure *ν*, such that, for some constant c>0 (the contraction rate),

$$\nu \left(\frac{\partial f}{\partial x}(x,t)\right)\leq -c,\forall x\in C,t\geq t_{0}.$$

The theory of contraction analysis states that, if a system is contracting as defined above, then all of its trajectories are incrementally exponentially stable (IES), as follows:

Lemma 4 **Theorem 1 in [13]**

*Suppose that*C*is a K-reachable forward-invariant subset of* Σ *and that the vector field defined by* f(x,t) *in* (4) *is infinitesimally contracting with contraction rate c therein. Then, for each two of* (4)*'s solutions* $x(t)=\phi (t,t_{0},x_{0})$ *and* $y(t)=\phi (t,t_{0},y_{0})$ *with* $x_{0},y_{0}\in C$ *we have that*

$$|x(t)-y(t)|\leq Ke^{-c(t-t_{0})}|x_{0}-y_{0}|,\forall t\geq t_{0}.$$

*As a result, if a system is contracting in a forward-invariant subset, then its solutions converge to a limiting trajectory.*

Remark 3 From Theorem 1 and Appendix B in [13], the contraction theorem is proved with $t_{0}=0$. Thus Definition 5 and Lemma 4 hold regardless of the value of $t_{0}$. The parameters *K* and *c* are also independent of $t_{0}$.

### 3.2 Theoretical results of contraction

By the idea in the proof of Theorem 2.2 in [27], we try to construct a sequence of functions $\left\{f^{m}(x,t)\right\}$ satisfying the conditions as follows, denoted by $C_{1}(\Sigma )$:

1. $f^{m}(x,t)$ is continuous and continuously differentiable w.r.t. *x* and continuous w.r.t. (x,t);
2. $\lim_{m\to \infty }d_{H}\left\{Graph(f^{m}(\Sigma ,t)),Graph(F(\Sigma ,t))\right\}=0$, for all $t\geq t_{0}$ and compact set $\Sigma \subset R^{n}$, where F(x,t)=K[f](x,t) is a set-valued function and $d_{H}$ stands for the Hausdorff metric. Graph(F) and $Graph(f^{m})$ are considered on $R^{n}\times [t_{0},+\infty )$, where $Graph(F(\{x\},t))=\{(x,t,y):x\in \Sigma ,t\geq t_{0},y\in F(x,t)\}$.
3. For compact set $\Sigma \subset R^{n}$, there exists a measure w(⋅), defined as w(Σ)=q(λ(Σ)), where *λ* denotes the Lebesgue measure and *q* is some measurable function (from $R^{+}$ to $R^{+}$), such that $\left|f^{m}(x,t)|\leq w(\Sigma \right)$ for all x∈Σ and $t\geq t_{0}$.

Notice that with Assumption 1 and Condition 2 in $C_{1}(\Sigma )$ above, we can find a subsequence of $\left\{f^{m}(x,t)\right\}$ satisfying Condition 3 in $C_{1}(\Sigma )$. So it is unnecessary to prove Condition 3 in the proof of Theorem 1 afterwards.

Lemma 5 *With Condition 2 of*$C_{1}(\Sigma )$*and*Assumption 1*, it can be proved that there exists a subsequence of*$\left\{f^{m}(x,t)\right\}$*satisfying Condition 3 above.*

Proof Under Assumption 1, |F(x,t)|≤a(t)|x|+b(t) holds for all $\left(x,t)\in R^{n}\times [t_{0},+\infty \right)$ with bounded functions a(t) and b(t), and there exists some continuous function $h(\cdot ):R^{+}\to R^{+}$ such that |F(x,t)−F(y,t)|≤h(|x−y|). It infers that |F(x,t)| is bounded for $\left(x,t)\in \Sigma \times [t_{0},+\infty \right)$, where Σ is a compact set. That is, |F(x,t)|≤m(Σ) holds for all x∈Σ and $t\geq t_{0}$ with some Lebesgue measurable function m(Σ).

With Condition 2 above, for each given ϵ>0, there exists large enough *N*, such that for all m≥N, x∈Σ and $t\geq t_{0}$, $|f^{m}(x,t)-F(x,t)|<\epsilon$ holds, which infers that $|f^{m}(x,t)|\leq m(\Sigma )+\epsilon$. Therefore, Lemma 5 is proved. □

Consider a sequence of differential systems as follows, with $\left\{f^{m}(x,t)\right\}$ satisfying conditions in $C_{1}(\Sigma )$:

(5) $${\overset{˙}{x}}^{m}=f^{m}(x^{m},t)$$

with $x^{m}(t_{0})=x^{0}$ for all $m\in N^{+}$.

Here we define the property of ‘uniform contraction’ for clearer expression afterwards.

Definition 6 We say that $\left\{f^{m}(x,t)\right\}$ is uniformly contracting in a K-reachable set $\Sigma \subset R^{n}$ w.r.t. a matrix measure ν(⋅,|⋅|) w.r.t. norm |⋅| if there exists α>0 such that

$$\nu \left(\frac{\partial f^{m}(x,t)}{\partial x},|\cdot |\right)\leq -\alpha$$

for all x∈Σ, $t\geq t_{0}$ and m=1,2,⋯.

We have the following main theorem.

Theorem 1 *Under*Assumption 1, Assumption 2*, let*$\left\{f^{m}(x,t)\right\}$*be the function sequence satisfying the conditions in*$C_{1}(\Sigma )$*and suppose that* Σ *is K-reachable, bounded and invariant for* (2) *and* (5)*. If* $\left\{f^{m}(x,t)\right\}$ *is uniformly contracting, the solutions of* (2) *exponentially converge towards each other in* Σ*.*

Proof From [15], under Assumption 1, Assumption 2, the solutions of the Cauchy problem of (2) and (5) exist and are unique respectively for $t\in R^{+}$.

For any two of the solutions of (2), x(t) and y(t) with different initial values $x(t_{0})=x^{0}$ and $y(t_{0})=y^{0}$, we construct solutions of (2) from sequences of solutions of (5), denoted by $x^{m}$ and $y^{m}$ respectively.

With $f^{m}$ satisfying condition $C_{1}(\Sigma )$, for each given *T*, $t_{0}\leq t\leq T$, we have $\left|x^{m}(t)|\leq M_{1}(x_{0},T\right)$, and $\left|{\overset{˙}{x}}^{m}(t)|\leq M_{2}(x_{0},T\right)$, where $M_{1}>0$, $M_{2}>0$ are bounded regarding $\left(x_{0},T\right)$. It is the same with $y^{m}(t)$. Thus $x^{m}(t)$ ($y^{m}(t)$) is uniformly bounded on $\left[t_{0},T\right]$, and ${\overset{˙}{x}}^{m}(t)$ (${\overset{˙}{y}}^{m}(t)$) is uniformly bounded on $\left[t_{0},T\right]$ as well.

Meanwhile, since $f^{m}$ is continuous and continuously differentiable with respect to *x* and continuous w.r.t. (x,t), $x^{m}(t)$ ($y^{m}(t)$) is continuously differentiable with respect to (x,t). Together with Condition 3 in $C_{1}(\Sigma )$, we conclude that $x^{m}(t)$ ($y^{m}(t)$), $m\in N^{+}$ are equicontinuous on $\left[t_{0},T\right]$.

Similar to Theorem 2.2 in [27], here we use Arzela-Ascoli lemma:

Lemma 6 *(Arzela-Ascoli Lemma) X is a compact set on*$R^{n}$*. If a sequence*${\left\{f_{n}\right\}}_{1}^{\infty }$*in*C(X)*is bounded and equicontinuous, then it has a uniformly convergent subsequence.*

We claim that, from Lemma 6 and diagonal selection principle, we can select a sub-sequence of ${\left\{x^{m}(t)\right\}}_{m\in N^{+}}$ and ${\left\{y^{m}(t)\right\}}_{m\in N^{+}}$ (still denoted by $x^{m}(t)$ and $y^{m}(t)$) such that $x^{m}(t)$ ($y^{m}(t)$) converges uniformly to a continuous function $x^{⁎}(t)$ ($y^{⁎}(t)$) on any compact interval of $\left[t_{0},+\infty \right)$. From Lemma 6, for all $k\in N^{+}$, we can find a subsequence ${\left\{x^{kj}(t)\right\}}_{j\in N^{+}}$ of ${\left\{x^{m}(t)\right\}}_{m\in N^{+}}$ such that $|x_{kj}(t)-x^{⁎}(t)|<\frac{1}{n}$ holds on the interval $\left[t_{0},t_{0}+k+1\right]$. Then we use diagonal selection principle and construct a new subsequence ${\left\{x^{kk}(t)\right\}}_{k\in N^{+}}$, which satisfies that ${\left\{x^{kk}(t)\right\}}_{k\in N^{+}}$ converges uniformly to a continuous function $x^{⁎}(t)$ on any compact interval of $\left[t_{0},+\infty \right)$.

The system ${\overset{˙}{x}}^{m}(t)=f^{m}(x,t)$ has a unique solution. $x^{m}(t)$ satisfies Lipschitz condition:

$$\left|x^{m}(t)-x^{m}(t^{\prime })|\leq L|t-t^{\prime }\right|$$

where $t,t^{\prime }\in [t_{0},T]$, *L* is a positive constant. This implies that $x^{⁎}(t)$ ($y^{⁎}(t)$) also satisfies Lipschitz condition, that is, ${\overset{˙}{x}}^{⁎}(t)$ (${\overset{˙}{y}}^{⁎}(t)$) exists and is measurable and bounded for any intervals of $\left[t_{0},+\infty \right)$.

We then claim that ${\overset{˙}{x}}^{m}(t)$ (${\overset{˙}{y}}^{m}(t)$) weakly converges to ${\overset{˙}{x}}^{⁎}(t)$ (${\overset{˙}{y}}^{⁎}(t)$) on the space $L_{1}([t_{0},T],R^{n})$ with following demonstrations.

$C_{0}^{\infty }([t_{0},T],R^{n})$ is dense in the Banach space $L^{\infty }([t_{0},T],R^{n})$, which is the conjugate space $L_{1}([t_{0},T],R^{n})$. Therefore, the following equation holds for each $p(t)\in C_{0}^{\infty }([t_{0},T],R^{n})$,

$$\int_{t_{0}}^{T}\langle {\overset{˙}{x}}^{m}(t)-{\overset{˙}{x}}^{⁎},p(t)\rangle dt=-\int_{t_{0}}^{T}\langle \overset{˙}{p}(t),x^{m}(t)-x^{⁎}\rangle dt.$$

With $\left\{{\overset{˙}{x}}^{m}(t)\right\}$ satisfying $\left|{\overset{˙}{x}}^{m}(t)|\leq M_{2}(x_{0},T\right)$, together with Lebesgue-dominant convergence theorem, it infers that

$$\lim_{m\to \infty }\int_{t_{0}}^{T}\langle {\overset{˙}{x}}^{m}(t)-{\overset{˙}{x}}^{⁎},p(t)\rangle dt=-\int_{t_{0}}^{T}\langle \overset{˙}{p}(t),\lim_{m\to \infty }x^{m}(t)-x^{⁎}(t)\rangle dt=0.$$

That is, $\left\{{\overset{˙}{x}}^{m}(t)\right\}$ weakly converges to ${\overset{˙}{x}}^{⁎}(t)$ on the space $L_{1}([t_{0},T],R^{n})$

By the Mazur's convexity theorem [27], there exists $a_{l}^{n}$ ($b_{n}^{l}$) with $\sum_{l=1}^{m}a_{l}^{m}=1$ ($\sum_{l=1}^{m}b_{l}^{m}=1$) such that, as m→∞, ${\overset{˙}{\overset{˜}{x}}}^{m}$ converges to ${\overset{˙}{x}}^{⁎}(t)$ almost everywhere on $\left[t_{0},T\right]$, where ${\overset{˜}{x}}^{m}(t)=\sum_{l=1}^{m}a_{l}^{m}x^{m}$. Notice that ${\overset{˜}{x}}^{m}$ is in the convex closure of $\left\{x^{m}\right\}$, therefore, ${\overset{˜}{x}}^{m}$ converges to $x^{⁎}$ uniformly. So it is with ${\overset{˜}{y}}^{m}(t)$, where ${\overset{˜}{y}}^{m}(t)=\sum_{l=1}^{m}b_{l}^{m}y^{m}$.

Review the second condition of $f^{m}$ in Condition $C_{1}(\Sigma )$. For $\Sigma \in R^{n}$, it holds that

$$\lim_{m\to \infty }d_{H}\left\{Graph(f^{m}(\Sigma ,t)),Graph(F(\Sigma ,t))\right\}=0,\forall t\geq t_{0},$$

where $d_{H}$ represents the Hausdorff metric and $Graph(F(x,t))=\{(x,t,y):x\in \Sigma ,t\in [t_{0},T],y\in F(x,t)\}$. With ${\overset{˙}{\overset{˜}{x}}}^{m}(t)$ in the convex closure of $\left\{f^{m}(x^{m},t)\right\}$, it infers that for any ϵ>0, there exists N>0 such that ${\overset{˙}{x}}^{m}(t)\in B(F(x^{⁎}(t),t),\epsilon )$ for all m>N and $x^{⁎},x^{m}\in \Sigma$, $t\in [t_{0},T]$. For a given vector norm |⋅|, B(x,δ)={y:|y−x|≤δ} stands for the *δ*-neighborhood of *x*.

Since *ϵ* can be arbitrarily small, it infers that ${\overset{˙}{x}}^{⁎}(t)\in F(x^{⁎}(t),t)$ with $x^{⁎}\in \Sigma$, which implies that the solution of (2) equals to $x^{⁎}(t)$ in Σ almost everywhere on $\left[t_{0},T\right]$. For x(t) should be continuous and $x^{⁎}$ is also continuous because ${\overset{˜}{x}}^{m}$ converges to $x^{⁎}$ uniformly on $\left[t_{0},T\right]$, $x^{⁎}$ is the solution of (2). So it is with $y^{⁎}(t)$.

Since the solution of (2) is unique, $x(t)=x^{⁎}(t)$ and $y(t)=y^{⁎}(t)$ almost every $t\geq t_{0}$. That is, $x^{m}$ converges to x(t) uniformly in any finite interval. Similar proof can be applied to $y^{m}(t)$ and y(t).

Since function sequence $\left\{f^{m}(x,t)\right\}$ is uniformly contracting, from Lemma 4, it can be shown that there exists some M=K>0 such that

$$\left|x^{m}(t)-y^{m}(t)|\leq Me^{-\alpha (t-t_{0})}|x^{0}-y^{0}\right|$$

for all $m\in N^{+}$ and $t\geq t_{0}$. For each given $t\geq t_{0}$, let $\epsilon (t)=3Me^{-\alpha (t-t_{0})}|x^{0}-y^{0}|$, there exists some $m_{0}(\epsilon ,t)$ with which $|x^{m}(t)-x(t)|\leq \epsilon /3$ and $|y^{m}(t)-y(t)|\leq \epsilon /3$ hold for $m\geq m_{0}(\epsilon ,t)$, which implies that

$$|x(t)-y(t)|\leq |x(t)-x^{m}(t)|+|y(t)-y^{m}(t)|+|x^{m}(t)-y^{m}(t)|\leq \epsilon =3Me^{-\alpha (t-t_{0})}|x^{0}-y^{0}|.$$

This completes the proof. □

Remark 4 One of the essential conditions for the contraction of Filippov system (4) is the existence and uniqueness of the solution of the underlying differential inclusion.

Remark 5 If (2) is regarded as a bimodal switched system, we can replace Assumption 2 with the conditions in Assumption 3 here in Theorem 1, which is applied in the following corollaries and examples. For more general switched systems defined as (3), by satisfying the conditions in Lemma 3 or proving the solution passes across the common boundary as illustrated in Corollary 1 in Sec. 10, Chapter 2 in [15], right uniqueness of Filippov solution for the switched system (3) can be proved in the whole domain.

Remark 6 In the proof of Theorem 1, the selection of the constants *M* and *α* are independent of $t_{0}$. So, the IES in Definition 3 can be achieved.

With Theorem 1, we have the following supplementary definition,

Definition 7 The Filippov system (2) is said to be contracting in the domain Σ w.r.t. the matrix measure *ν* if there exists a continuously and differentiable function sequence $\left\{f^{m}(x,t)\right\}$ with condition $C_{1}$ such that $f^{m}$ is uniformly contracting in domain Σ with respect to *ν*.

Herein, we present several tips for how to construct the function sequence $\left\{f^{m}(x,t)\right\}$ for validation of the condition in Theorem 1 to guarantee that incremental stability can be obtained by the constraint $\nu (\frac{\partial f^{m}(x,t)}{\partial x})<0$. We consider two scenarios as follows.

**Switched system**. Consider the switched system (3) with the notations as above with necessary specifications and assume that the conditions for the existence and uniqueness of the Cauchy problem are satisfied. Let σ(⋅):R→[0,1] be continuous, non-decreasing, and differentiable,^1^ with σ(ρ)=0 when $\rho \leq -\frac{1}{2}$, and σ(ρ)=1 when $\rho \geq \frac{1}{2}$. We also assume that each function $f_{i}(x,t)$ for each region $R_{i}$ is well-defined in the whole region. The core idea is to construct a continuous and differentiable function sequence that is a convex combination of functions $f_{i}(x,t)$ in the neighborhood region of the hypersurfaces $S_{i}$ as well as their intersections in order to converge (in graph) to the set-valued map K(f)(x,t).

First, we consider a simple example of switched system with a single switching hypersurface $S_{0}=\{(x,t):\phi_{0}(x,t)=0\}$ as follows:

(6) $$\overset{˙}{x}=f(x,t)=\left\{ \begin{array}{cc} f_{1}(x,t), & \phi_{0}(x,t)>0,\\ f_{2}(x,t), & \phi_{0}(x,t)<0, \end{array}\right.$$

then we construct a sequence of systems ${\overset{˙}{x}}^{m}=f^{m}(x^{m},t)$ as the convex combination of $f_{1,2}(x,t)$, where

(7) $$f^{m}(x,t)=\sigma \left(\frac{\phi_{0}(x,t)}{\delta }\right)f_{1}(x,t)+\left[1-\sigma \left(\frac{\phi_{0}(x,t)}{\delta }\right)\right]f_{2}(x,t),\;\;\delta =\frac{1}{m}.$$

**Figure 1:**
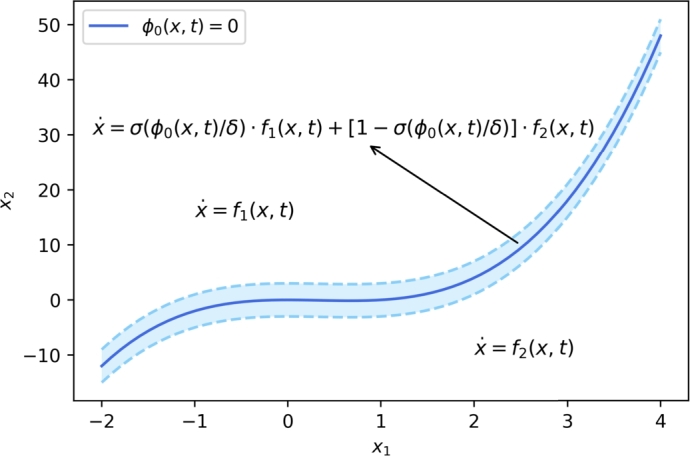
A simple schematic diagram for switched system (6) and the construction method.

A simple schematic diagram is shown in Fig. 1. It can be shown that the sequence of functions $\left\{f^{m}(x,t)\right\}$ formulated as (7) is to be proved to approach the set-valued map as follows:

$$F(x,t)=K[f](x,t)=\left\{ \begin{array}{cc} f_{1}(x,t), & \phi_{0}(x,t)>0,\\ {⋂}_{\delta >0}\bar{co}\{f_{1}(B^{+}(x,\delta ),t),f_{2}(B^{-}(x,\delta ),t)\}, & \phi_{0}(x,t)=0,\\ f_{2}(x,t), & \phi_{0}(x,t)<0, \end{array}\right.$$

as m→∞, where $B^{+}(x,\delta )=B(x,\delta )\cap \{x:\phi_{0}(x,t)>0\}$ and $B^{-}(x,\delta )=B(x,\delta )\cap \{x:\phi_{0}(x,t)<0\}$. Note

$$\underset{\delta >0}{⋂}\bar{co}\{f_{1}(B^{+}(x,\delta ),t),f_{2}(B^{-}(x,\delta ),t)\}\;=\{af_{1}(x,t)+(1-a)f_{2}(x,t):a\in [0,1]\},$$

for each (x,t) with $\phi_{0}(x,t)=0$.

For more complex circumstances, consider the switched system (3) with the notations as above and denote the whole set of switching hypersurfaces by $\left\{S_{i}\right\}$. We here propose an iterative way to construct the function sequence $\left\{f^{m}(x,t)\right\}$ similar to the processing above.

Generally speaking, let g(x,t) be the switched function composed of continuous and differentiable functions $g_{j}(x,t)$, j=1,⋯,P, defined in each regions $Q_{j}$, the functions and $S_{i_{⁎}}$ be certain hypersurface with switching function $\phi_{i_{⁎}}(x,t)=0$. We firstly construct two functions $g{|}_{i_{⁎}^{\pm }}^{\delta }$ that coincide with g(x,t) in the two subregions $Q_{\delta }^{+}=\{(x,t):\phi_{i_{⁎}}(x,t)>\delta /2\}$ and $Q_{\delta }^{+}=\{(x,t):\phi_{i_{⁎}}(x,t)<-\delta /2\}$ and are continuous and differentiable on the neighborhood region of $S_{i_{⁎}}$: $Q=\{(x,t):-\delta /2\leq \phi_{i_{⁎}}(x,t)\leq \delta /2\}$. Then, we can construct the function sequences as the convex combination of $g{|}_{i_{⁎}^{\pm }}^{\delta }$ as follows:

(8) $$g^{\delta }(x,t)=\sigma \left(\frac{\phi_{i_{⁎}}(x,t)}{\delta }\right)g{|}_{i_{⁎}^{+}}^{\delta }(x,t)+\left[1-\sigma \left(\frac{\phi_{i_{⁎}}(x,t)}{\delta }\right)\right]g{|}_{i_{⁎}^{-}}^{\delta }(x,t).$$

Let δ=1/m. So, we can get the function sequence that is continuous and differentiable in the neighborhood of $S_{i_{⁎}}$ and take values as the convex combination of the values of g(x,t) from the both sides of $S_{i_{⁎}}$. Hence, we iteratively consider all switching hypersurfaces in the same way and can obtain a continuous and differentiable function sequences that are convex combinations of f(x,t) from both sides near each switching hypersurfaces to validate the conditions $C_{1}(\Sigma )$. An explicit and direct way to construct $g{|}_{i_{⁎}^{\pm }}^{\delta }$ can be formulated as follows.

(9) $$g{|}_{i_{⁎}^{+}}^{\delta }=g_{j}(x,t)\;\mathrm{for}\;j=\text{a}rgmin_{k:\;Q_{k}\subset \{(x,t):\phi_{i_{⁎}}(x,t)>0\}}\beta ((x,t),Q_{k})g{|}_{i_{⁎}^{-}}^{\delta }=g_{p}(x,t)\;\mathrm{for}\;p=\text{a}rgmin_{q:\;Q_{q}\subset \{(x,t):\phi_{i_{⁎}}(x,t)<0\}}\beta ((x,t),Q_{q}).$$

Here, $\beta ((x,t),V)=\inf_{u\in V}|(x,t)-u{|}_{\infty }$ for some region *V* with $|\cdot {|}_{\infty }$ denoting the $L_{\infty }$-norm. Note that $g{|}_{i_{⁎}^{\pm }}^{\delta }$ are defined on $(\Sigma \times R^{+})\setminus \left[{⋃}_{j\neq i_{⁎}}S_{j}\right]⋃\left\{(x,t):|\phi_{i_{⁎}}(x,t)|<\delta /2\right\}$.

In detail, we are in the stage to present the iterative method to construct the function sequence $\left\{f^{m}(x,t)\right\}$ as follows. Let N={1,2,⋯,N} be the index set of the switching hypersurfaces $S_{i}$, i=1,⋯,N.

**Figure 2:**
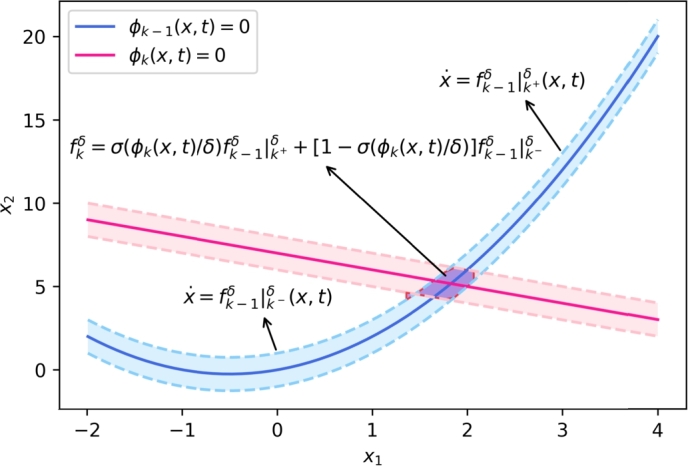
A simple schematic diagram for the recursive construction method for switched system (3) with *S*_*k*−1_ and *S*_*k*_ intersecting.
Step 3: Loop through Step 2 and update the functions until $I_{N}=N$. Then we construct the sequence of functions as $f^{m}(x,t)=f_{N}^{\delta }(x,t)$ by taking δ=1/m.

Step 1: We start with a single switching hypersurface $S_{1}$ and let $I_{1}=\{1\}$ be the index set for the hypersurfaces that is considered in the iterative way. By the method mentioned in the paragraph above and Eq. (8), we are able to construct the functions with respect to the origin switching function f(x,t):
  $$f_{1}^{\delta }=\sigma \left(\frac{\phi_{1}(x,t)}{\delta }\right)f{|}_{1^{+}}^{\delta }(x,t)+\left[1-\sigma \left(\frac{\phi_{1}(x,t)}{\delta }\right)\right]f{|}_{1^{-}}^{\delta }(x,t)$$
  with $f{|}_{1^{\pm }}(x,t)$ defined above, for example by Eq. (9).
Step 2: Iteratively, assume that for the hypersurfaces $\left\{S_{1},S_{2},\cdots ,S_{k-1}\right\}$ with the index set $I_{k-1}=\{1,\cdots ,k-1\}$, for k≥1, the functions $f_{k-1}^{\delta }$ are well constructed an continuous and differentiable on $(U\times R^{+})\setminus {⋃}_{i\leq k-1}S_{i}$. Consider a new hypersurface $S_{k}$ with $\phi_{k}(x,t)$. For $I_{k}=I_{k-1}⋃\{k\}$, let $f_{k-1}^{\delta }{|}_{k^{\pm }}^{\delta }(x,t)$ be continuous and differentiable extension from $f_{k-1}^{\delta }$ in the *δ*-neighborhood of $S_{k}$ as mentioned above. A direct and explicit version is given by (9). Then we give
  (10) $$f_{k}^{\delta }(x,t)=\sigma \left(\frac{\phi_{k}(x,t)}{\delta }\right)f_{k-1}^{\delta }{|}_{k^{+}}^{\delta }(x,t)+\left[1-\sigma \left(\frac{\phi_{k}(x,t)}{\delta }\right)\right]f_{k-1}^{\delta }{|}_{k^{-}}^{\delta }(x,t).$$
  A simple schematic diagram for two intersecting hypersurfaces $S_{k-1}$ and $S_{k}$ is illustrated in Fig. 2.

**Composite function of discontinuous functions**. Here, we consider the case that discontinuity of the right-hand functions is caused by certain discontinuous function of lower-dimensions, formulated as follows.

(11) $$\overset{˙}{x}=f(x,t)=h(g(x),t)$$

where $x={\left(x_{1},x_{2},...,x_{n}\right)}^{⊤}$ is the state vector, $h(y,t):R^{+}\times R^{k}\to R^{n}$ is continuous w.r.t. y,t, and $g(x):R^{n}\to R^{k}$ gives the discontinuity. Suppose that there is a sequence of functions $\left\{g^{m}(x)\right\}$ satisfying Condition 1 and 3 in $C_{1}$, and $\lim_{m\to \infty }d_{H}\left\{Graph(g^{m}(\Sigma ,t)),Graph(G(\Sigma ,t))\right\}=0$, for all t≥0 and any compact set $\Sigma \subset R^{n}$, similar to Condition 2 in $C_{1}$, where G(x,t)=K[g](x,t).

Then we construct $\left\{f^{m}(x,t)\right\}$ as follows,

$$f^{m}(x,t)=h(g^{m}(x),t),\;\delta =\frac{1}{m}.$$

It is easy to prove that the sequence of functions $f^{m}(x,t)$ approach the right-hand side of (11) in the sense of set-valued map as *δ* converges to zero.

Remark 7 We have presented two special scenarios on how to construct function sequence $f^{m}(x,t)$ towards satisfying the conditions of Theorem 1. However, we highlight that validation the conditions of Theorem 1 including $C_{1}(\Sigma )$ should be further conducted.

## 4 Application examples

### 4.1 Switched differential systems

Here we take a simple case of switched system (3) as follows:

(12) $$\overset{˙}{x}=f(x,t)=\left\{ \begin{array}{cc} f_{1}(x,t) & z^{⊤}x>0\\ f_{2}(x,t) & z^{⊤}x<0 \end{array}\right.$$

where $z\in R^{n}$, $x\in R^{n}$, with the initial default time $t_{0}=0$, and *z* defines the switching hyperplane with $z^{⊤}z=1$.

On the discontinuous surface $z^{⊤}x=0$, the right-hand side of the system should be consistent with the differential inclusion of the system according to (2) in the following analysis. Thus, for $\left(x,t)\in \{z^{⊤}x=0\right\}$, it should be guaranteed that $\overset{˙}{x}(t)=f(x,t)\in K[f](x,t)$, and

$$K[f](x,t)=\underset{\delta >0}{⋂}\underset{\mu (S)=0}{⋂}\bar{co}\{f(B(x,\delta )\setminus S,t)\}=\underset{\delta >0}{⋂}\bar{co}\{f_{1}(B^{+}(x,\delta ),t),f_{2}(B^{-}(x,\delta ),t)\},$$

where $B^{+}(x,\delta )=\{z^{⊤}x>0\}\cap B(x,\delta )$, $B^{-}(x,\delta )=\{z^{⊤}x<0\}\cap B(x,\delta )$. That is, on $\left\{z^{⊤}x=0\right\}$, f(x,t) should be in $\left\{af_{1}(x,t)+(1-a)f_{2}(x,t):a\in [0,1]\right\}$. Also, we need to guarantee the existence and uniqueness of the system's solution according to the conclusions in [15], [23].

We have the following result:

Corollary 1 *With the notations in*Definition 6*, suppose that the continuously differentiable vector field defined by*$f_{1}(x,t)$*and*$f_{2}(x,t)$*are contracting (with parameter α) in their domain respectively for matrix measure ν with respect to norm*|⋅|*. Suppose*$L(t)=\max\{|\lim_{x\to \overset{\to }{0}}f_{1}(x,t)|,|\lim_{x\to \overset{\to }{0}}f_{2}(x,t)|\}$*is bounded for*$t\in R^{+}$*and*$M=\frac{1}{\alpha }\sup_{t\in R^{+}}L(t)$*.*

*If there exists*δ>0*, such that*

(13) $$\lim_{z^{⊤}x\to 0^{+}}z^{⊤}f_{1}(x,t)<0\;\;or\;\;\lim_{z^{⊤}x\to 0^{-}}z^{⊤}f_{2}(x,t)>0,$$

(14) $$\nu ([f_{1}(x,t)-f_{2}(x,t)]\cdot z^{⊤})\leq 0,$$

*holds for*$x\in \{x:-\frac{\delta }{2}\leq z^{⊤}x\leq \frac{\delta }{2}\}$*, where*δ>0*can be arbitrarily small, then it can be concluded that the Filippov solutions of system*(12)*globally exponentially converge towards each other almost everywhere in*$\Sigma =\{x\in R^{n}:|x|\leq M\}$*.*

Proof We need first to prove that the Cauchy problem of (12) has a unique solution. Note that the Dini derivative of |x(t)|

(15) $$D^{+}|x(t)|\leq \max\{\nu (\frac{\partial f}{\partial x})\}|x(t)|+\max\{|\lim_{x\to \overset{\to }{0}}f_{1}(x,t)|,|\lim_{x\to \overset{\to }{0}}f_{2}(x,t)|\}\leq -\alpha |x(t)|+\max\{|\lim_{x\to \overset{\to }{0}}f_{1}(x,t)|,|\lim_{x\to \overset{\to }{0}}f_{2}(x,t)|\}.$$

This implies that with *M* valued above, there exists some T>0 such that |x(T)|≤M holds for all t≥T. Let Σ denote $\left\{x\in R^{n}:|x|\leq M\right\}$. The inequality above can also be rewritten as $D^{+}|x(t)-x(0)|\leq -\alpha |x(t)-x(0)|+{\bar{\lim}}_{x\to x(0)}|f(x(0),t)|$, which means our proof here is applicable in the neighborhood of its initial state.

We first prove that system (12) satisfies Assumption 1, which ensures the existence of solution of (12). With x∈Σ and F(x,t)=K[f](x,t) is bounded and closed, from (15), it is obvious that one can find functions a(t) and b(t) that are bounded on $R^{+}$, satisfying

|F(x,t)|≤a(t)|x|+b(t).

What's more, since F(x,t) is bounded, there exists continuous function $h(\cdot ):R^{+}\to R^{+}$, such that |F(x,t)−F(y,t)|≤h(|x−y|) always holds for x,y∈Σ. So system (12) satisfies Assumption 1 here.

Furthermore, when it comes to Assumption 3, condition (13) is sufficient to guarantee uniqueness of (12)'s solution. Therefore, the existence and uniqueness of the solution of (12) is proved under the conditions proposed above.

Note that $f_{1}$ and $f_{2}$ are contracting as given, that is, $\nu (\frac{\partial f_{1}}{\partial x})<0$ for $z^{⊤}x>0$ and $\nu (\frac{\partial f_{2}}{\partial x})<0$ for $z^{⊤}x<0$ holds for matrix measure ν(⋅,|⋅|).

Then we construct a sequence of maps as (7). For the system ${\overset{˙}{x}}^{m}=f^{m}(x^{m},t)$, where

$$f^{m}(x,t)=\sigma \left(\frac{z^{⊤}x}{\delta }\right)f_{1}(x,t)+\left[1-\sigma \left(\frac{z^{⊤}x}{\delta }\right)\right]f_{2}(x,t),\;\;\delta =\frac{1}{m},$$

with σ(⋅) defined as

(16) $$\sigma (s)=\left\{ \begin{array}{cc} 0, & s<-1/2,\\ s+1/2, & s\in [-1/2,1/2],\\ 1, & s>1/2. \end{array}\right.$$

We have

$$\frac{\partial f^{m}(x,t)}{\partial x}=\left[f_{1}(x,t)-f_{2}(x,t)\right]\cdot \frac{z^{⊤}}{\delta }+\sigma \left(\frac{z^{⊤}x}{\delta }\right)\frac{\partial f_{1}(x,t)}{\partial x}+\left[1-\sigma \left(\frac{z^{⊤}x}{\delta }\right)\right]\frac{\partial f_{2}(x,t)}{\partial x}.$$

Under the condition (14) that

$$\nu ([f_{1}(x,t)-f_{2}(x,t)]\cdot z^{⊤})\leq 0$$

holds for each *x* that satisfies $|z^{⊤}x|\leq \frac{\delta }{2}$, it can be shown that $\frac{\partial f^{m}(x,t)}{\partial x}<-\alpha$ holds for all sufficient large *m*. From Theorem 1, the Filippov solutions with different initial values of (12) globally exponentially converge towards each other almost everywhere in Σ. The corollary is proved. □

Corollary 2 *If given that system* (12) *has a unique solution, then only the condition* $\nu ([f_{1}(x,t)-f_{2}(x,t)]z^{⊤})\leq 0$ *is necessary to prove the exponential stability in* Corollary 1 *, that is, condition* (13) *is no longer needed.*

Remark 8 Notice that the switching hypersurface $\left\{x:z^{⊤}x=0\right\}$ here is linear. When it comes to a system formulated like (3) with nonlinear discontinuities, a similar conclusion can be proposed and proved as well.

Remark 9 From Proposition 3 of [20], it can be proved that for any two vectors $x,y\in R^{n}$, x,y≠0 and for any norm, we have that $\nu (xy^{⊤})\geq 0$.

So, more generally, together with Remark 8, we have

Corollary 3 *With the notations and definitions in*(3)*, together with the condition that*$B(S_{i},\epsilon )$*and*$B(S_{j},\epsilon )$*have no intersection for all*i≠j*and some*ϵ>0*, suppose that*$f_{G_{i}^{+}}$*and*$f_{G_{i}^{-}}$*are contracting for all*i=1,2,...,N*, and for*$t\in R^{+}$*, the Cauchy problem of*(3)*has a unique solution in domain* Σ*, if there exists* δ>0*, such that*

$$\nu ([f_{G_{i}^{+}}(x,t)-f_{G_{i}^{-}}(x,t)]\cdot \frac{\partial \phi_{i}(x,t)}{\partial x})=0$$

*holds for each x that satisfies* $|\phi_{i}(x,t)|<\delta$*, δ can be arbitrarily small,* i=1,2,...,N*, then the system* (3) *is globally exponential stable almost everywhere in* Σ*.*

The detailed proof for Corollary 3 is omitted here, which is similar to the proof of Corollary 1.

Remark 10 Similar approach above has been employed in Theorem 11 in [20]. In [20], under the assumption that (12) satisfies the conditions for the existence and uniqueness of a Caratheodory solution, the conclusion is that, with $f^{+}$ and $f^{-}$ contracting in their domains and $\nu ([f^{+}(x,t)-f^{-}(x,t)]\cdot {▽}_{x}\phi_{i}(x,t))=0$ in the neighborhood of the switching hypersurface $S_{i}$, the bimodal Filippov system is incrementally exponentially stable in a *K*-reachable set with a specific convergence rate, which is consistent with Corollary 3.

### 4.2 Switched linear systems

Here we take a switched linear differential system, which is also called a discontinuous piecewise affine (PWA) system for example. More detailed conditions for exponential stability can be obtained here. Consider the PWA system as follows:

(17) $$\overset{˙}{x}=\left\{ \begin{array}{cc} Ax+a, & z^{⊤}x>0,\\ Bx+b, & z^{⊤}x<0, \end{array}\right.$$

with some matrices $A,B\in R^{n,n}$, vectors $a,b,z\in R^{n}$, and *z* defines the switching hyperplane, $z^{⊤}z=1$. It is guaranteed that on the discontinuous surface $z^{⊤}x=0$, the right-hand side f(x,t)∈{k(Ax+a)+(1−k)(Bx+b):k∈[0,1]}, which is consistent with the differential inclusion in the following proof, and the system has a unique solution according to the assumptions above.

Here we similarly construct a smooth approximation of the discontinuous right-hand side. Specific conditions for exponential stability are expounded as follows.

Corollary 4 *Suppose that there exists a positive constant α, such that*ν(A)≤−α*and*ν(B)≤−α*holds for some matrix measure*ν(⋅,|⋅|)*.*

*Let*V=−max⁡(|a|,|b|)/max⁡(ν(A),ν(B))*, and β be the transaction coefficient with*$\left|y{|}_{2}\leq \beta |y\right|$*for all*$y\in R^{n}$*, where*$|\cdot {|}_{2}$*denotes the 2-norm. If*

(18) $$\beta V\sqrt{|{\left(A-B\right)}^{⊤}z{|}_{2}^{2}-{\left(z^{⊤}(A-B)z\right)}^{2}}+z^{⊤}(a-b)<0,$$

(19) $$\nu ([(A-B)x+a-b]z^{⊤})<\alpha \delta$$

*holds for*|x|≤V*and*$|z^{⊤}x|<\frac{\delta }{2}$*, where*δ>0*can be arbitrarily small, then the Filippov solutions of*(17)*exponentially converge towards each other in compact set*Σ={x:|x|≤V}*.*

Proof Here we first prove the existence and uniqueness of the Filippov solution. Note that Dini derivative of |x(t)|

$$D^{+}|x(t)|\leq \max\{\nu (A),\nu (B)\}|x(t)|+\max\{|a|,|b|\}.$$

Let Σ={x:|x|≤V}. This implies the boundedness of solutions with initial states in Σ. The inequality above can be written as $D^{+}|x(t)-x(0)|\leq \max\{\nu (A),\nu (B)\}|x(t)-x(0)|+\max\{|a|,|b|\}$, which means our proof here is also applicable on the neighborhood of the initial state.

Meanwhile, let $Q=\beta V\sqrt{|{\left(A-B\right)}^{⊤}z{|}_{2}^{2}-{\left(z^{⊤}(A-B)z\right)}^{2}}$. From KKT conditions for Lagrange function, it can be proved that the maximum value of the objective function in the following optimization problem:

$$\left\{ \begin{array}{cc} \max_{x} & z^{⊤}[(A-B)x+(a-b)]\\ s.t.\; & |x{|}_{2}\leq \beta V,|z^{⊤}x|\leq \delta /2 \end{array}\right.$$

can be written as $Q+z^{⊤}(a-b)+O(\delta )$ as δ→0. So, under the condition (18), one can see that $z^{⊤}[(Ax+a)-(Bx+b)]<0$, which satisfies Assumption 3. This guarantees that system (17) has a unique solution.

Similarly, we construct the following sequence of functions:

$$f^{m}(x)=\sigma \left(\frac{z^{⊤}x}{\delta }\right)(Ax+a)+\left[1-\sigma \left(\frac{z^{⊤}x}{\delta }\right)\right](Bx+b),\;\delta =\frac{1}{m}$$

with σ(⋅) in the form of (16), which can be shown to approach the corresponding set-valued map of (17) as δ→0. The system ${\overset{˙}{x}}^{m}=f^{m}(x^{m})$ is bounded with the bound *M* as well.

In the case $|z^{⊤}x|\leq \delta /2$, we have

$$\frac{\partial f^{m}(x)}{\partial x}=\sigma (z^{⊤}x/\delta )A+(1-\sigma (z^{⊤}x/\delta ))B+\frac{1}{\delta }[Ax+a-Bx-b]z^{⊤},\delta =\frac{1}{m}.$$

Then under the condition (19) that

$$\nu ([(A-B)x+a-b]\cdot z^{⊤})<\alpha \delta$$

holds for $|x{|}_{2}\leq \beta V,|z^{⊤}x|\leq \delta /2$, which satisfies $\nu (\frac{f^{m}(x)}{\partial x})<0$, guarantees the exponential stability, as proved in Theorem 1. □

From Corollary 4 above, we find it consistent with the following corollary, which has already been proposed in Theorem 2 in [29]:

Corollary 5 *System*(17)*is incrementally exponentially stable if there exists a positive definite matrix*$J=J^{⊤}>0$*, a vector*$g\in R^{n}$*and a number*γ∈(0,1)*such that*

*1.* $JA+A^{⊤}J<0$*,*$JB+B^{⊤}J<0$*;*
*2.* $A-B=gz^{⊤}$*;*
*3.* J(a−b)=−γz*.*

The detailed proof is added in Appendix B.

Remark 11 In Corollary 5, under the circumstance that γ=0 which implies a=b, the conditions above infer that the right-hand side of system (17) is continuous. Under the circumstance that γ=1, it indicates that the discontinuity may occur only due to the shift terms *a* and *b*.

Furthermore, we can take matrix measure defined w.r.t. some other norms. For example, when we take norm |⋅| defined for matrix $A=(a_{ij})$ as $|A|=|\xi^{-1}A\xi {|}_{1}$, where $\xi =diag\{\xi_{1},...,\xi_{n}\}$, which implies that $\nu_{\xi ,1}(A)=\max_{j}\{a_{jj}+\sum_{i\neq j}|\xi_{i}\xi_{j}^{-1}a_{ij}|\}$, we have the following conclusion:

Corollary 6 *Suppose there exists matrix*$\xi =diag\{\xi_{1},...,\xi_{n}\}$*, such that*

$$\nu_{\xi ,1}(A)=\max_{j}\{a_{jj}+\sum_{i\neq j}|\xi_{i}\xi_{j}^{-1}a_{ij}|\}\leq -\alpha ,\nu_{\xi ,1}(B)=\max_{j}\{b_{jj}+\sum_{i\neq j}|\xi_{i}\xi_{j}^{-1}b_{ij}|\}\leq -\alpha$$

*for some*α>0*. Let*$V=-\max(|a|,|b|)/\max(\nu_{\xi ,1}(A),\nu_{\xi ,1}(B))$*, and β be the transaction coefficient with*$\left|y{|}_{2}\leq \beta |y\right|$*for all*$y\in R^{n}$*. If*

$$\beta V\sqrt{|{\left(A-B\right)}^{⊤}z{|}_{2}^{2}-{\left(z^{⊤}(A-B)z\right)}^{2}}+z^{⊤}(a-b)<0,\nu_{\xi ,1}([(A-B)x+a-b]z^{⊤})<\alpha \delta ,$$

*holds for*|x|≤V*and*$|z^{⊤}x|<\frac{\delta }{2}$*, where*δ>0*can be arbitrarily small, then system*(17)*is exponentially stable in compact set*Σ={x:|x|≤V}*.*

### 4.3 Switched systems with hypersurfaces $S_{i}$ intersecting

**Figure 3:**
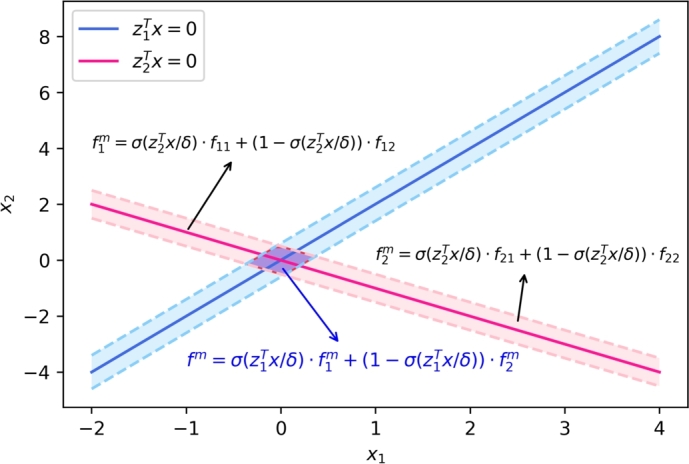
A simple schematic diagram when two switching hypersurfaces intersect each other.

In this section, we consider system (3), with the switching hypersurfaces intersecting each other. For simplicity, we here study the system as follows for example,

(20) $$\overset{˙}{x}=f(x,t)=\left\{ \begin{array}{cc} f_{11}(x,t), & z_{1}^{⊤}x>0,z_{2}^{⊤}x>0,\\ f_{12}(x,t), & z_{1}^{⊤}x>0,z_{2}^{⊤}x<0,\\ f_{21}(x,t), & z_{1}^{⊤}x<0,z_{2}^{⊤}x>0,\\ f_{22}(x,t), & z_{1}^{⊤}x<0,z_{2}^{⊤}x<0, \end{array}\right.$$

where $z_{1},z_{2}\in R^{n}$, $x\in R^{n}$, and $z_{1},z_{2}$ define the switching hyperplanes with $z_{1}^{⊤}z_{1}=1$ and $z_{2}^{⊤}z_{2}=1$. And on the discontinuous surfaces, the right-hand side should be consistent with the differential inclusion of the system in the following analysis. A simple schematic diagram is shown in Fig. 3.

Corollary 7 *Suppose that system* (20) *satisfies* Assumption 1, Assumption 3 *, and with the matrix measure ν with respect to norm* |⋅| *,* $\nu (\frac{\partial f(x,t)}{\partial x})<-\alpha$ *holds on its continuous regions. If*

(21) $$\begin{array}{c} \nu [(f_{11}(x,t)-f_{21}(x,t))z_{1}^{⊤}]<\alpha \delta ,\\ \nu [(f_{12}(x,t)-f_{22}(x,t))z_{1}^{⊤}]<\alpha \delta \end{array}$$

*holds for* $|z_{1}^{⊤}x|<\frac{\delta }{2}$ *, and*

(22) $$\begin{array}{c} \nu [(f_{11}(x,t)-f_{12}(x,t))z_{2}^{⊤}]<\alpha \delta ,\\ \nu [(f_{21}(x,t)-f_{22}(x,t))z_{2}^{⊤}]<\alpha \delta \end{array}$$

*holds for* $|z_{2}^{⊤}x|<\frac{\delta }{2}$ *, then the Filippov solutions of* (20) *can be proved exponentially stable.*

Proof Similar to Corollary 1, it can be shown that the Cauchy problem of (20) has a unique solution. Then we construct a sequence of maps with the construction method above for switched systems as in Eq. (9) and (10). We first construct the following continuous functions on $\left\{(x,t):|z_{1}^{⊤}x|\neq 0\right\}$:

$$f_{z_{2}}^{m}(x,t)=\;\left\{ \begin{array}{cc} f_{1}^{m}(x,t)=\sigma \left(\frac{z_{2}^{⊤}x}{\delta }\right)f_{11}(x,t)+\left[1-\sigma \left(\frac{z_{2}^{⊤}x}{\delta }\right)\right]f_{12}(x,t), & z_{1}^{⊤}x>0,\\ f_{2}^{m}(x,t)=\sigma \left(\frac{z_{2}^{⊤}x}{\delta }\right)f_{21}(x,t)+\left[1-\sigma \left(\frac{z_{2}^{⊤}x}{\delta }\right)\right]f_{22}(x,t), & z_{1}^{⊤}x<0, \end{array}\right.$$

with σ(⋅) defined as (16). Then, $f^{m}(x,t)$ is designed as follows,

$$f^{m}(x,t)=\sigma \left(\frac{z_{1}^{⊤}x}{\delta }\right)f_{1}^{m}(x,t)+\left[1-\sigma \left(\frac{z_{1}^{⊤}x}{\delta }\right)\right]f_{2}^{m}(x,t).$$

It can be shown that $f^{m}(x,t)$ is continuous and approaches the set-valued map F(x,t)=K[f](x,t). The derivative of $f^{m}(x,t)$ w.r.t. *x*,

$$\frac{\partial f^{m}(x,t)}{\partial x}=\sigma \left(\frac{z_{1}^{⊤}x}{\delta }\right)\sigma \left(\frac{z_{2}^{⊤}x}{\delta }\right)f_{11}(x,t)+\sigma \left(\frac{z_{1}^{⊤}x}{\delta }\right)\left[1-\sigma \left(\frac{z_{2}^{⊤}x}{\delta }\right)\right]f_{12}(x,t)+\sigma \left(\frac{z_{2}^{⊤}x}{\delta }\right)\left[1-\sigma \left(\frac{z_{1}^{⊤}x}{\delta }\right)\right]f_{21}(x,t)+\left[1-\sigma \left(\frac{z_{1}^{⊤}x}{\delta }\right)\right]\left[1-\sigma \left(\frac{z_{2}^{⊤}x}{\delta }\right)\right]f_{22}(x,t),+\left\{\sigma \left(\frac{z_{2}^{⊤}x}{\delta }\right)(f_{11}(x,t)-f_{21}(x,t)\right)+\left[1-\sigma \left(\frac{z_{2}^{⊤}x}{\delta }\right)\right](f_{12}(x,t)-f_{22}(x,t))\}\frac{z_{1}^{⊤}}{\delta }+\left\{\sigma \left(\frac{z_{1}^{⊤}x}{\delta }\right)(f_{11}(x,t)-f_{12}(x,t)\right)+\left[1-\sigma \left(\frac{z_{1}^{⊤}x}{\delta }\right)\right](f_{21}(x,t)-f_{22}(x,t))\}\frac{z_{2}^{⊤}}{\delta }$$

needs to satisfy that its measure $\nu (\frac{\partial f^{m}(x,t)}{\partial x})$ should be less than zero as δ→0. which is satisfied by (21) and (22). □

### 4.4 Hopfield neural networks with discontinuous activation functions

Hopfield neural networks with discontinuous activation functions can be modeled as follows [18], [30],

(23) $$\overset{˙}{x}=-D(t)x+T(t)g(x)+J,$$

where $x={\left(x_{1},x_{2},...,x_{n}\right)}^{⊤}$ is the state vector of the neural network. For $t\in R^{+}$, $D(t)=diag\{d_{1}(t),...,d_{n}(t)\}$ with $d_{i}(\cdot )$ modeling self-inhibition of *i*-th neuron. $T(t)=(T_{ij}(t))\in R^{n,n}$ is the connection matrix, and $J=(J_{1},J_{2},...,J_{n})\in R^{n,n}$ is input vector, $g(x)={\left(g_{1}(x_{1}),g_{2}(x_{2}),...,g_{n}(x_{n})\right)}^{⊤}$ with $g_{i}(\cdot )$ modeling the non-linear input-output activation of *i*-th neuron.

Here, we list the following assumptions, denoted by Condition $C_{2}$:

1. there exists $\overset{˜}{D}=diag\{D_{1},D_{2},...,D_{n}\}$, $D_{i}>0$, such that $d_{i}(\cdot )$ is continuous and $\frac{d_{i}(\zeta_{1})-d_{i}(\zeta_{2})}{\zeta_{1}-\zeta_{2}}\geq D_{i}$ for i=1,2,...,n and any $\zeta_{1}\neq \zeta_{2}$.
2. $g_{i}(\cdot )$ is non-decreasing and in every compact set of R, each $g_{i}(\cdot )$ has only finite discontinuous points. Therefore, in any compact set in R, except finite points $\rho_{k}$, where there exist finite right and left limits $g_{i}(\rho_{k}^{+})$ and $g_{i}(\rho_{k}^{-})$ with $g_{i}(\rho_{k}^{+})>g_{i}(\rho_{k}^{-})$, $g_{i}(\cdot )$ is continuous.

For matrix $A=(a_{ij})$, we define the matrix measure as $\nu_{\xi ,1}(A)=\max_{j}[a_{jj}+\sum_{i\neq j}|\xi_{i}\xi_{j}^{-1}a_{ij}|]$ with respect to norm $|\cdot {|}_{\xi ,1}$, $|A|=|\xi A\xi^{-1}{|}_{1}$, where $\xi =diag\{\xi_{1},...,\xi_{n}\}$. We can prove the following Corollary:

Corollary 8 *Suppose that the solution of the Cauchy problem of*(23)*exists and is unique for*$t\in R^{+}$*, and system*(23)*satisfies Condition*$C_{2}$*above, and there exists a positive diagonal matrix*$\xi =diag\{\xi_{1},...,\xi_{n}\}$*, such that*

(24) $$\nu_{\xi ,1}(T)=\max_{j}\{T_{jj}(t)+\sum_{i\neq j}|\xi_{i}\xi_{j}^{-1}T_{ij}(t)|\}\leq 0$$

*holds for*$t\in R^{+}$*. Then the Filippov solutions of*(23)*exponentially converge towards each other.*

Proof Under the assumption that the solution of system (23) exists and is unique for $t\in R^{+}$, by the way of (11), we construct the continuous system as follows:

$$\overset{˙}{x}=f^{\delta }(x,t)=-D(t)x+T(t)\overset{˜}{g}(x)+J,$$

where $\overset{˜}{g}(x)={\left({\overset{˜}{g}}_{1}(x_{1}),{\overset{˜}{g}}_{2}(x_{2}),...,{\overset{˜}{g}}_{n}(x_{n})\right)}^{⊤}$. For each *i* and each discontinuous point, denoted by $x_{0i}$ below, if $x_{i}\notin [x_{0i}-\frac{\delta }{2},x_{0i}+\frac{\delta }{2}]$, let ${\overset{˜}{g}}_{i}(x_{i})=g_{i}(x_{i})$, and if $x_{i}\in [x_{0i}-\frac{\delta }{2},x_{0i}+\frac{\delta }{2}]$, let

$$\overset{˜}{g_{i}}(x_{i})=\frac{g_{i}(x_{0i}+\frac{\delta }{2})-g_{i}(x_{0i}-\frac{\delta }{2})}{\delta }[x_{i}-x_{0i}+\frac{\delta }{2}]+g_{i}(x_{0i}-\frac{\delta }{2}).$$

It is easy to prove that the sequence of functions $\left\{f^{\delta }(x)\right\}$ approaches the right-hand side of (23) in the sense of set-valued map as *δ* converges to zero.

Let ${\overset{¯}{g}}_{i}(x_{0i},\delta )=\frac{g_{i}(x_{0i}+\frac{\delta }{2})-g_{1}(x_{0i}-\frac{\delta }{2})}{\delta }$. Here we denote by *G*,

$$G=\frac{\partial \overset{˜}{g}(x)}{\partial x}{|}_{x=(x_{01},x_{02},...,x_{0n})}=diag\{{\overset{¯}{g}}_{1}(x_{01},\delta ),{\overset{¯}{g}}_{2}(x_{02},\delta ),...,{\overset{¯}{g}}_{n}(x_{0n},\delta )\}.$$

That is, for $x_{i}\in [x_{0i}-\frac{\delta }{2},x_{0i}+\frac{\delta }{2}]$, i=1,2,...,n, we have

$$\frac{\partial f^{\delta }(x)}{\partial x}=-D(t)+T(t)G.$$

Here we take the matrix measure $\nu_{\xi ,1}(\cdot )$, we need to ensure that $\nu_{\xi ,1}(-D(t)+T(t)G)<0$ for all $t\in R^{+}$. With Condition $C_{2}$ above, we already have $\nu_{\xi ,1}(-D(t))<-\min_{j}\{D_{j}\}$. Meanwhile, we have

$$\nu_{\xi ,1}(T(t)G)=\max_{j}\{T_{jj}(t){\overset{¯}{g}}_{j}(x_{0j},\delta )+\sum_{i\neq j}|T_{ij}(t){\overset{¯}{g}}_{j}(x_{0j},\delta )|\}.$$

As for T(t)G, because $g_{i}(\cdot )$ is non-decreasing, it is obvious that *G* is a non-negative diagonal matrix.

With the condition (24), there exists a positive diagonal matrix *ξ*, such that $\max_{j}\{T_{jj}(t)+\sum_{i\neq j}|\xi_{i}\xi_{j}^{-1}T_{ij}(t)|\}\leq 0$. Thus,

$$\nu_{\xi ,1}(T(t)G)\leq \max_{j}\{T_{jj}(t)+\sum_{i\neq j}|T_{ij}|\}\cdot \min_{k}{\overset{¯}{g}}_{k}(x_{0k},\delta )\leq 0.$$

Therefore, we have

$$\nu_{\xi ,1}(-D(t)+T(t)G)\leq \nu_{\xi ,1}(-D(t))+\nu_{\xi ,1}(T(t)G)<-\min_{k}\{D_{k}\},$$

and from Theorem 1, the Filippov solutions of (23) exponentially converge towards each other. □

### 4.5 Sliding mode control

In this part, the application of sliding mode control (SMC) [31] is discussed. Here we denote $x={\left(x_{1},x_{2},...,x_{n}\right)}^{⊤}\in R^{n}$, so we have the following system:

(25) $$\overset{˙}{x}=f(x,t)=\left\{ \begin{array}{c} f_{1}(x,t),\;s(x)>0,\\ f_{2}(x,t),\;s(x)<0, \end{array}\right.$$

with $\nu (\frac{\partial f_{1}(x,t)}{\partial x})<-\alpha$ and $\nu (\frac{\partial f_{2}(x,t)}{\partial x})<-\alpha$, α>0. The stable manifold is a (n−1)-dimensional hyperplane {s(x)=0}.

The definition of “stable manifold” is that, if the Filippov trajectory of (25) reaches the hyperplane {s(x)=0} in finite time, the solution of (25) will converge to origin in finite time as well. Here we aim to find the sufficient conditions for $f_{1}(x,t)$, $f_{2}(x,t)$ and s(x) to control the trajectory to converge to the stable manifold and thus x(t) converges to zero in finite time.

To illustrate the application to sliding mode control, here we take a simple case for example from Section 1.3 in [32], with the positive integer ρ≥2, the *ρ*-th order system is as follows.

$$\left\{ \begin{array}{cc} {\overset{˙}{x}}_{i} & =x_{i+1},\;\;1\leq i\leq \rho -1,\\ {\overset{˙}{x}}_{\rho } & =u(x,t). \end{array}\right.$$

For simplicity, let ρ=2, we consider the second order system:

(26) $$\left\{ \begin{array}{cc} {\overset{˙}{x}}_{1} & =x_{2},\\ {\overset{˙}{x}}_{2} & =u(x,t), \end{array}\right.$$

with $x_{1}$ converging to zero in finite time if its trajectory reaches $z_{1}x_{1}+z_{2}{\overset{˙}{x}}_{1}=0$, where $z_{1}z_{2}>0$ and $z_{1}^{2}+z_{2}^{2}=1$. Then we seek for the qualified control law u(t) to guarantee the trajectory of (26) converges to origin in finite time.

Define coordinate with respect to stable manifold:

(27) $$s=z^{⊤}{\left(x_{1},{\overset{˙}{x}}_{1}\right)}^{⊤}=0,$$

where $z={\left(z_{1},z_{2}\right)}^{⊤}$, such that $x_{1}$ is stable if s=0. We have the conclusion as follows.

Corollary 9 *Suppose system*(26)*satisfies*Assumption 1*and there exists*α>0*, such that*

(28) $$\nu (\left(\begin{array}{cc} 0\; & 1\\ \frac{\partial u(x,t)}{\partial x_{1}}\; & \frac{\partial u(x,t)}{\partial x_{2}} \end{array}\right))<-\alpha$$

*in the continuous regions. And for*$|z^{⊤}x|<\frac{\delta }{2}$*, δ can be arbitrarily small, together with matrix measure*ν(⋅,|⋅|)*is as defined in*Definition 2*, if the following conditions are satisfied,*

(29) $$\nu (\left(\begin{array}{cc} 0\; & 0\\ z_{1}(u_{1}(x,t)-u_{2}(x,t))\; & z_{2}(u_{1}(x,t)-u_{2}(x,t)) \end{array}\right))<\alpha \delta ,z_{1}x_{2}+z_{2}u_{1}(x,t)<0,\;when\;z^{⊤}x\to 0^{+},z_{1}x_{2}+z_{2}u_{2}(x,t)>0,\;when\;z^{⊤}x\to 0^{-}$$

*where*$u(x,t)=u_{1}(x,t)$*for*$s=z^{⊤}x>0$*, and*$u(x,t)=u_{2}(x,t)$*when*$s=z^{⊤}x<0$*, then it can be proved that the Filippov solutions will finally converge to the origin.*

The corollary above is easily obtained by constructing a sequence of functions satisfying Condition $C_{1}$, together with Theorem 1.

Proof Here we denote $x={\left(x_{1},x_{2}\right)}^{⊤}$, so we have the following system:

$$\overset{˙}{x}=f(x,t)=\left(\begin{array}{c} x_{2}\\ u(x,t) \end{array}\right).$$

Obviously, we need f(x,t) to be a switched function, with $u(x,t)=u_{1}(x,t)$ for $s=z^{⊤}x>0$, and $u(x,t)=u_{2}(x,t)$ when $s=z^{⊤}x<0$. Let

$$u^{\delta }(x,t)=\sigma (\frac{z^{⊤}x}{\delta })u_{1}(x,t)+[1-\sigma (\frac{z^{⊤}x}{\delta })]u_{2}(x,t)$$

for $|z^{⊤}x|<\frac{\delta }{2}$. Thus, from Theorem 1, $u_{1}(x,t)$ and $u_{2}(x,t)$ need to satisfy the following conditions:

In the continuous region,

$$\nu (\frac{\partial f(x,t)}{\partial x})=\nu (\left(\begin{array}{cc} 0\; & 1\\ \frac{\partial u(x,t)}{\partial x_{1}}\; & \frac{\partial u(x,t)}{\partial x_{2}} \end{array}\right))<-\alpha ,$$

and for $|z^{⊤}x|<\frac{\delta }{2}$,

(30) $$\nu (\left(\begin{array}{cc} 0\; & 1\\ \frac{\partial u^{\delta }(x,t)}{\partial x_{1}}\; & \frac{\partial u^{\delta }(x,t)}{\partial x_{2}} \end{array}\right))<0\;z_{1}x_{2}+z_{2}u_{1}(x,t)<0,\;z^{⊤}x\to 0^{+},z_{1}x_{2}+z_{2}u_{2}(x,t)>0,\;z^{⊤}x\to 0^{-},$$

where matrix measure ν(⋅,‖⋅‖) is defined in Definition 2. Consider (30), let $v(x,t)=u_{1}(x,t)-u_{2}(x,t)$, $r_{1}=\nu (\left(\begin{array}{cc} 0\; & 1\\ \frac{\partial u_{1}}{\partial x_{1}}\; & \frac{\partial u_{1}}{\partial x_{2}} \end{array}\right))$, $r_{2}=\nu (\left(\begin{array}{cc} 0\; & 1\\ \frac{\partial u_{2}}{\partial x_{1}}\; & \frac{\partial u_{2}}{\partial x_{2}} \end{array}\right))$. We have

$$\nu (\left(\begin{array}{cc} 0\; & 1\\ \frac{\partial u^{\delta }}{\partial x_{1}}\; & \frac{\partial u^{\delta }}{\partial x_{2}} \end{array}\right))\leq \nu (\left(\begin{array}{cc} 0\; & 0\\ z_{1}v\; & z_{2}v \end{array}\right))+\sigma (\frac{z^{⊤}x}{\delta })r_{1}+[1-\sigma (\frac{z^{⊤}x}{\delta })]r_{2}.$$

Thus, under the condition (29), Eq. (30) is satisfied. From Theorem 1, it is proved that the Filippov solutions of (26) exponentially converge towards each other. Considering a trajectory with its initial point satisfying (27), which obviously converges to origin in finite time, the trajectories with other initial inputs will finally converge to origin as well. The corollary is proved. □

Remark 12 For sliding mode control problems, a stricter condition is necessary to guarantee the solutions of system (25) converge to the stable manifold in finite time. Together with the conditions above in Corollary 9, the system needs to satisfy that there exists β>0 such that

$$z_{1}x_{2}+z_{2}u_{1}(x,t)<-\beta \;\text{when}\;z^{⊤}x\to 0^{+},z_{1}x_{2}+z_{2}u_{2}(x,t)>\beta \;\text{when}\;z^{⊤}x\to 0^{-}.$$

Since the system is incrementally exponentially stable according to Corollary 9, the trajectory will converge to the *δ*-neighborhood of the origin in finite time, where *δ* can be arbitrarily small. Then with the new condition above, if the trajectory reaches the *δ*-neighborhood of the stable manifold $z^{⊤}x=0$, where *δ* is small enough, it is obvious that the trajectory will converge the stable manifold in finite time.

Remark 13 The spectral radius of the matrix may help deal with the conditions proposed in Corollary 9. In the continuous region, it is obvious that if the spectral radius of matrix $I+h\frac{\partial f(x,t)}{\partial x}$, denoted by $\rho (I+h\frac{\partial f(x,t)}{\partial x})$, is less than 1, then there exists a matrix norm |⋅|, such that $|I+h\frac{\partial f(x,t)}{\partial x}|\leq \rho (I+h\frac{\partial f(x,t)}{\partial x})+\alpha \leq 1$, that is, $\nu (\frac{\partial f(x,t)}{\partial x})<-\alpha$, where α>0 can be arbitrarily small.

$\rho (I+h\frac{\partial f(x,t)}{\partial x})=\max R\left(\lambda (I+h\frac{\partial f(x,t)}{\partial x})\right)$, where λ(⋅) represents the matrix's eigenvalues and R(z) refers to the absolute value of real *z* or the mode of imaginary *z*. It is the same with inequality (28) and (29), whose sufficient conditions are calculated through the spectral radius as $R\left(1+h\frac{\partial u}{\partial x_{2}}+h\sqrt{{\left(\frac{\partial u}{\partial x_{2}}\right)}^{2}+4\frac{\partial u}{\partial x_{1}}}\right)<1$, and $z_{2}(u_{1}(x,t)-u_{2}(x,t))<0$ respectively.

## 5 Numerical experiments

In this section, several numerical experiments are presented to help illustrate the main theorem and corollaries proposed above.

### 5.1 Example 1: one-dimensional switched systems

We take the one-dimensional switched system as an example to illustrate the main theorem. Consider the following switched system:

$$\overset{˙}{x}=-x-sign(x)$$

with the sign function sign, which can be formulated as a differential inclusion: $\overset{˙}{x}\in F(x)$ with

$$F(x)=\left\{ \begin{array}{cc} -x+1 & x<0\\ -x-[-1,1] & x=0\\ -x-1 & x>0 \end{array}\right.\;.$$

So, it can be seen that $\Sigma_{K}=\{x:|x|\leq K\}$ for any K≥1 is invariant for this differential inclusion. Consider the following continuously differentiable function sequence:

$$f^{m}(x)=-x-\frac{2}{\pi }\arctan(mx)$$

which can be shown to approach F(x) in graph as m→∞ and the differential systems: $\overset{˙}{x}=f^{m}(x)$ with the Jacobian: $df^{m}(x)/dx=-1-(2/\pi )(m/(1+m^{2}x^{2}))$, which are uniformly contracting with α=−1. Hence, we can prove that $\overset{˙}{x}\in F(x)$ is contracting as well.

### 5.2 Example 2: switched nonlinear system

Consider the following switched nonlinear system. Through the criteria proposed in Corollary 1, it can be proved that the following system is exponential stable, which is verified by numerical experiment.

(31) $$\overset{˙}{x}=f(x,t)=\left\{ \begin{array}{cc} xz^{⊤}x-x+z, & z^{⊤}x<0,\\ -xz^{⊤}x-x-z, & z^{⊤}x>0, \end{array}\right.$$

where $z,x\in R^{d}$, $z^{⊤}z=1$, and on the surface $\left\{z^{⊤}x=0\right\}$, $\overset{˙}{x}(t)\in \{(1-2a)z-x:k\in [0,1]\}$.

Then calculate the derivative of the right side,

$$\frac{\partial f}{\partial x}=\left\{ \begin{array}{cc} Iz^{⊤}x+xz^{⊤}I-I, & z^{⊤}x<0,\\ -Iz^{⊤}x-xz^{⊤}I-I, & z^{⊤}x>0. \end{array}\right.$$

It can be proved that there exists some matrix measure *ν* w.r.t. |⋅| (e.g. 2-norm) such that $\nu (\frac{\partial f}{\partial x})\leq -1$ (that is, $\nu (Iz^{⊤}x+xz^{⊤}I)>0$ if $z^{⊤}x>0$ and $\nu (Iz^{⊤}x+xz^{⊤}I)<0$ if $z^{⊤}x<0$).

Note the Dini derivative of |x(t)|,

$$D^{+}|x(t)|\leq \nu (\frac{\partial f}{\partial x})|x|+|z|$$

This implies that there exists some |x(t)|≤|z| for all t≥T and some T>0. Let $\Sigma =\{x\in R^{3}:|x|\leq |z|\}$.

It can be verified that the system has a unique solution, satisfying the condition (13) for

$$z^{⊤}(f_{1}(x,t)-f_{2}(x,t))=-2{\left(z^{⊤}x\right)}^{2}-2<0$$

and also satisfying condition (14) for when δ→0,

$$\nu ((f_{1}(x,t)-f_{2}(x,t))\cdot z^{⊤})=(-2xz^{⊤}xz^{⊤}-2zz^{⊤})\leq \nu (-2(\delta xz^{⊤})-2zz^{⊤})<0.$$

**Figure 4:**
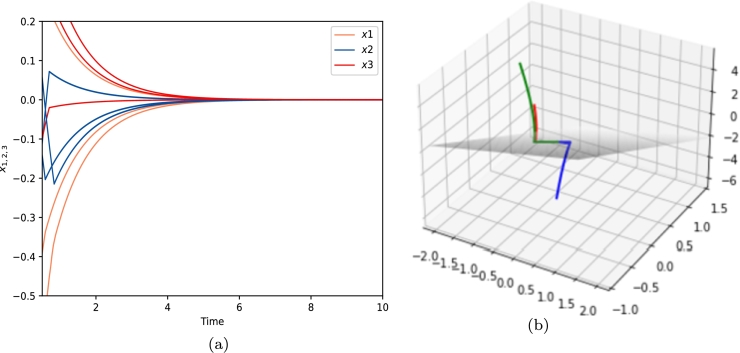
Convergent Dynamics of (31). This figure shows the dynamics of *x*_1_, *x*_2_ and *x*_3_ with initial values in $\left\{x\in R^{3}:|x|\leq |z|\right\}$ (randomly selected of uniform distribution).

Therefore, according to the conclusion in Corollary 1, the nonlinear switched system (31) is exponentially stable. Fig. 4 shows the convergent dynamics of (31).

Since $x(t)={\left(0,0,0\right)}^{⊤}$ is a special solution of system (31), the solutions will finally converge to the origin as seen in Fig. 4. We find that the dynamics of system (31) converges to the discontinuous surface in finite time under the effect of the discontinuous surface since

$$\frac{\partial (z^{⊤}x)}{\partial t}=\left\{ \begin{array}{cc} -z^{⊤}x(z^{⊤}x+1)-1<-1, & z^{⊤}x>0,\\ z^{⊤}x(z^{⊤}x-1)+1>1, & z^{⊤}x<0. \end{array}\right.$$

On the surface $\left\{z^{⊤}x=0\right\}$, according to the constraints proposed above, we have $\overset{˙}{x}(t)\in \{(1-2a)z-x:a\in [0,1]\}$, which implies that x(t) is converging towards the coordinate origin at an exponential speed.

### 5.3 Example 3: sliding mode control

We take the following system for instance:

(32) $$\overset{˙}{x}=f(x,t)=\left(\begin{array}{c} x_{2}\\ u(x,t) \end{array}\right),$$

where u(x,t) is a switched function with switching surface $\left\{z^{⊤}x=0\right\}$. Let $u(x,t)=u_{1}(x,t)$ when $z^{⊤}x>0$, and $u(x,t)=u_{2}(x,t)$ when $z^{⊤}x<0$.

**Figure 5:**
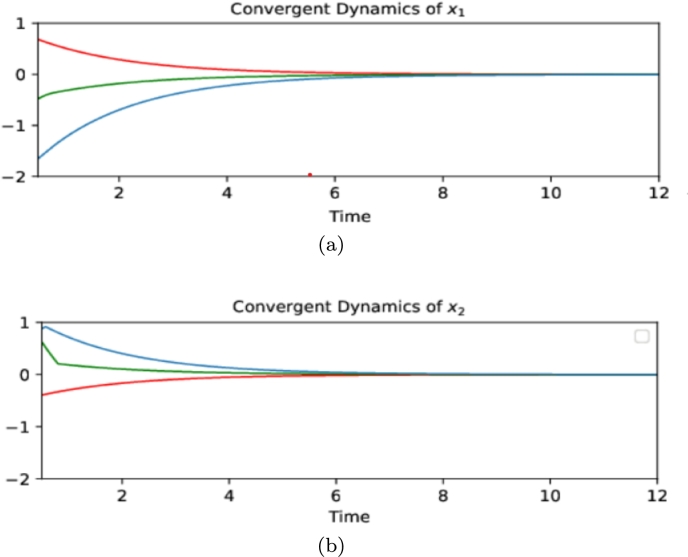
Convergent Dynamics of *x*_1_ and *x*_2_ in (32). This figure illustrates the dynamics of *x*_1_ and *x*_2_ respectively in (32) with different initial values.

**Figure 6:**
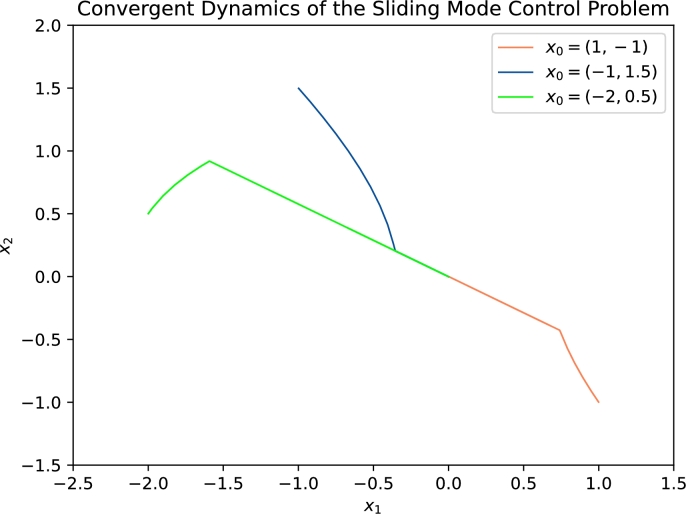
Convergent trajectory of (32). This figure intuitively shows the convergent trajectory of *x*_1_ and *x*_2_ in (32) with initial values (1,−1)^⊤^, (−1,0.5)^⊤^, (−2,0.5)^⊤^ respectively.

Let $u_{1}(x,t)=-\frac{z_{1}}{z_{2}}(x_{2}+1)$ and $u_{2}(x,t)=-\frac{z_{1}}{z_{2}}(x_{2}-1)$ together with $z=(\frac{1}{2},\frac{\sqrt{3}}{2})$, then $u_{1}(x,t)$ and $u_{2}(x,t)$ satisfy the conditions above in Corollary 9 in Section 4.5. u(x,t) is a qualified control law which insures the stability. Fig. 5 and Fig. 6 show the convergent dynamics with initial values $x_{0}={\left(1,-1\right)}^{⊤}$, ${\left(-1,1.5\right)}^{⊤}$, ${\left(-2,0.5\right)}^{⊤}$, respectively.

## 6 Conclusion and future work

In conclusions, we proposed a general criterion on the conditions for exponential incremental stability of a class of dynamical systems with discontinuous right-hand. We formulated the corresponding Filippov system for the discontinuous system and constructed a sequence of contracting dynamical systems with continuous right-hand sides to approximate the Filippov system. With the uniform contraction of the sequence of continuous dynamical systems, the incremental stability of the Filippov system is proved in Theorem 1. To demonstrate the power of the present theory, we took several types of discontinuous dynamical systems as examples and found the criteria is efficient by selecting different matrix measures, compared with the previous ones. Meanwhile, the application to sliding mode control (SMC) problems is attached and a new train of thought is proposed, which may help construct the control law. Furthermore, how it performs compared with classical Lyapunov method for more complicate control problem related to Filippov system needs further investigation.

## Declarations

### Author contribution statement

Lu Wenlian: conceived and designed the experiments; performed the experiments; contributed reagents, materials, analysis tools or data.

Lang Yingying: performed the experiments; analyzed and interpreted the data; contributed reagents, materials, analysis tools or data; wrote the paper.

### Funding statement

Wenlian Lu was supported by 10.13039/501100012166National Key R&D Program of China [2018AAA0100303], 10.13039/501100001809National Natural Science Foundation of China [62072111], Shanghai Municipal Science and Technology Major Project [2018SHZDZX01].

### Data availability statement

No data was used for the research described in the article.

### Declaration of interests statement

The authors declare no competing interests.

### Additional information

No additional information is available for this paper.

## Figures and Tables

**Figure 7:**
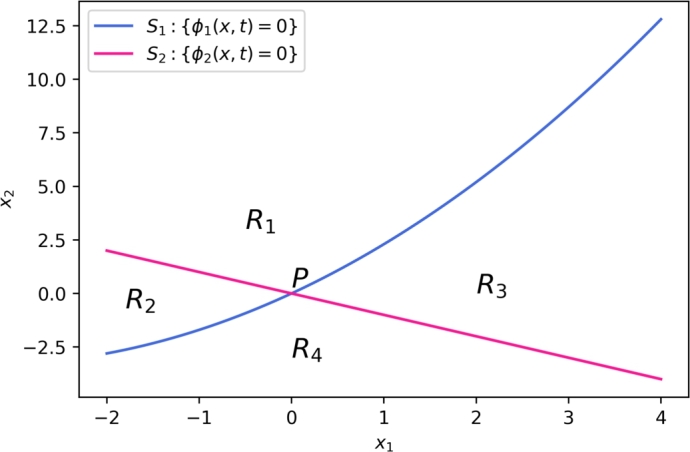
A simple schematic diagram for the notations when two switching hypersurfaces intersect each other.

## Appendix A An example: try to prove uniqueness under given conditions with Lemma 3

Here we take a model with two switching surfaces intersecting each other and research the conditions for uniqueness for illustration.

As seem in Fig. 7,

(33) $$\overset{˙}{x}=f_{i}(x,t),x\in R_{i}\subset R^{2},i=1,2,3,4,$$

where $R_{1}=\{\phi_{1}(x,t)>0\}\cap \{\phi_{2}(x,t)>0\}$, $R_{2}=\{\phi_{1}(x,t)>0\}\cap \{\phi_{2}(x,t)<0\}$, $R_{3}=\{\phi_{1}(x,t)<0\}\cap \{\phi_{2}(x,t)>0\}$, $R_{4}=\{\phi_{1}(x,t)<0\}\cap \{\phi_{2}(x,t)<0\}$. Let $f^{R_{k}}$ be the limiting values of the function *f* at the point (x,t) on the boundary of $R_{k}$ from the region $R_{k}$. Then let $f_{N_{i}}^{R_{k}}$ be the corresponding projections of the vectors onto the normal to $S_{i}$ at point $(x,t)\in S_{i}$. Assume that $S_{1}$ and $S_{2}$ intersect each other at one point $P=S_{1}\cap S_{2}$.

We claim that, under the condition that Assumption 3 is satisfied for points $(x,t)\in S_{1}\cup S_{2}$, and following conditions, denoted by Condition $C_{\text{(33)}}$ are satisfied at the intersection of the switching surfaces $P=S_{1}\cap S_{2}$,

1. ($f_{N_{1}}^{R_{1}}<0$ and $f_{N_{1}}^{R_{2}}<0$) or ($f_{N_{1}}^{R_{3}}>0$ and $f_{N_{1}}^{R_{4}}>0$),
2. ($f_{N_{2}}^{R_{1}}<0$ and $f_{N_{2}}^{R_{3}}<0$) or ($f_{N_{2}}^{R_{2}}>0$ and $f_{N_{2}}^{R_{4}}>0$),

uniqueness occurs for the whole domain. The detailed proof is complemented as follows.

For two regions $R_{i}$ and $R_{j}$, i,j∈{1,2,3,4}, if $R_{i}$ and $R_{j}$ are on the same side of the switching surface $S_{1}$ (or $S_{2}$), the uniqueness of the system's solution occurs on $R_{i}\cup R_{j}\cup (\partial R_{i}\cap \partial R_{j})$, with Assumption 3 satisfied w.r.t. the switching surface $S_{2}$ (or $S_{1}$), according to Lemma 2, where ∂*R* means the boundary of region *R*. Problem comes when we consider the intersection of the two switching surfaces $P=S_{1}\cap S_{2}$, which is also the common boundary of regions $R_{1}$ and $R_{4}$, regions $R_{2}$ and $R_{3}$.

We here take regions $R_{1}$ and $R_{4}$ for instance, and they have the common boundary *P*. According to Condition $C_{\text{(33)}}$ given above, we have the following conditions at the point *P*,

1. $f_{N_{1}}^{R_{1}}<0$ or $f_{N_{1}}^{R_{4}}>0$,
2. $f_{N_{2}}^{R_{1}}<0$ or $f_{N_{2}}^{R_{4}}>0$.

It can be proved that if $f_{N_{1}}^{R_{1}}<0$ and $f_{N_{1}}^{R_{4}}<0$ (or $f_{N_{1}}^{R_{1}}>0$ and $f_{N_{1}}^{R_{4}}>0$) at point *P*, the solutions pass from $R_{1}\cup R_{2}$ into $R_{3}\cup R_{4}$ (or from $R_{3}\cup R_{4}$ into $R_{1}\cup R_{2}$) in the neighborhood of *P* by virtue of Corollary 1 of Lemma 2 in Sec. 10, Chapter 2 in [15], thus we just need to analyze the conditions for uniqueness according to the switching surface $S_{2}$ with Lemma 2, which is consistent to the conditions above. It is the same if $f_{N_{2}}^{R_{1}}<0$ and $f_{N_{2}}^{R_{4}}<0$ (or $f_{N_{2}}^{R_{1}}>0$ and $f_{N_{2}}^{R_{4}}>0$) at the point *P*.

Then we need to consider the circumstance that $f_{N_{1}}^{R_{4}}-f_{N_{1}}^{R_{1}}>0$ and $f_{N_{2}}^{R_{4}}-f_{N_{2}}^{R_{1}}>0$. Here we need to use Lemma 3 (Theorem 1 in Sec. 10, Chapter 2 in [15]) for detailed analysis.

For $x\in R_{1}$ and $y\in R_{4}$ in the neighborhood of (z,t)∈P, from the boundedness of $\partial f/\partial x_{i}$ it follows that

(34) $$|f(x,t)-f^{R_{1}}(z,t)|\leq l|x-z|,\;\;\;\;|f(y,t)-f^{R_{4}}(z,t)|\leq l|y-z|.$$

Similar to the proof of Theorem 2 in Sec. 10, Chapter 2 in [15], it follows from the inequalities $f_{N_{1}}^{R_{4}}-f_{N_{1}}^{R_{1}}>0$ and $f_{N_{2}}^{R_{4}}-f_{N_{2}}^{R_{1}}>0$ that

(35) $$(x-z)(f^{R_{1}}(z,t)-f^{R_{4}}(z,t))\leq 0.$$

This inequality remains valid also if the vector x−z is replaced by the vector x−y which has the same direction. Adding the derived inequality (35) to the one following from (34)

$$(x-y)(f(x,t)+f^{R_{4}}(z,t)-f^{R_{1}}(z,t)-f(y,t))<l|y-x{|}^{2}.$$

That is,

$$(x-y)(f(x,t)-f(y,t))<l|y-x{|}^{2}.$$

It is the same with $R_{2}$ and $R_{3}$.

Thus from Lemma 3, the uniqueness occurs in the neighborhood of (z,t) in domain. The proof is completed.

## Appendix B Proof of Corollary 5 by virtue of Corollary 4

Corollary 4. Suppose that there exists a positive constant *α*, such that ν(A)≤−α and ν(B)≤−α holds for some matrix measure ν(⋅,|⋅|).

Let V=−max⁡(|a|,|b|)/max⁡(ν(A),ν(B)), and *β* be the transaction coefficient with $\left|y{|}_{2}\leq \beta |y\right|$ for all $y\in R^{n}$, where $|\cdot {|}_{2}$ denotes the 2-norm. If

(36) $$\beta V\sqrt{|{\left(A-B\right)}^{⊤}z{|}_{2}^{2}-{\left(z^{⊤}(A-B)z\right)}^{2}}+z^{⊤}(a-b)<0,\nu ([(A-B)x+a-b]z^{⊤})<\alpha \delta$$

holds for |x|≤V and $|z^{⊤}x|<\frac{\delta }{2}$, where δ>0 can be arbitrarily small, then the Filippov solutions of (17) exponentially converge towards each other in compact set Σ={x:|x|≤V}.

Corollary 5. System (17) is incrementally exponentially stable if there exists a positive definite matrix $J=J^{⊤}>0$, a vector $g\in R^{n}$ and a number γ∈(0,1) such that

1. $JA+A^{⊤}J<0$, $JB+B^{⊤}J<0$;
2. $A-B=gz^{⊤}$;
3. J(a−b)=−γz.

Proof With matrix measure defined with respect to norm |⋅|, for matrix *Q*, $|Q|=|J^{\frac{1}{2}}QJ^{-\frac{1}{2}}{|}_{2}$, where *J* is a positive definite symmetric matrix, we can find that

$$\nu (w\cdot z^{⊤})=\lambda_{max}(\frac{J^{\frac{1}{2}}wz^{⊤}J^{-\frac{1}{2}}+J^{-\frac{1}{2}}zw^{⊤}J^{\frac{1}{2}}}{2})=\frac{1}{2}(|J^{\frac{1}{2}}w{|}_{2}|J^{-\frac{1}{2}}z{|}_{2}+z^{⊤}w).$$

Let x=u+kz, where u⊥z and $|k|\leq \frac{\delta }{2}$. Let h=(A−B)u, ∀u⊥z, then we have

$$2\nu (w\cdot z^{⊤})=|J^{-\frac{1}{2}}z{|}_{2}\cdot |J^{\frac{1}{2}}(A-B)x+J^{\frac{1}{2}}(a-b){|}_{2}+z^{⊤}(A-B)x+z^{⊤}(a-b)=|J^{\frac{1}{2}}(h+k(A-B)z+a-b){|}_{2}\cdot |J^{-\frac{1}{2}}z{|}_{2}+z^{⊤}h+kz^{⊤}(A-B)z+z^{⊤}(a-b).$$

From (36) we need $\frac{1}{\delta }\nu (w\cdot z^{⊤})\leq \alpha$ holds for $|z^{⊤}x|<\frac{\delta }{2}$, which infers that $\frac{1}{\delta }|J^{\frac{1}{2}}(h+a-b){|}_{2}|J^{-\frac{1}{2}}z{|}_{2}+\frac{1}{\delta }z^{⊤}h+\frac{1}{\delta }z^{⊤}(a-b)$ should be upper bounded even if *δ* goes to zero, i.e.,

$$z^{⊤}h+|J^{-\frac{1}{2}}z{|}_{2}|J^{\frac{1}{2}}(h+a-b){|}_{2}+z^{⊤}(a-b)=0.$$

Since *u* can take any value that satisfies u⊥z, it implies that $h=(A-B)u=\overset{\to }{0}$ together with $|J^{-\frac{1}{2}}z{|}_{2}|J^{\frac{1}{2}}(a-b){|}_{2}+z^{⊤}(a-b)=0$, that is, there exists γ>0, such that $J^{\frac{1}{2}}(a-b)=-\gamma J^{-\frac{1}{2}}z$, i.e., J(a−b)=−γz. Meanwhile, from ν(A)<0 we conclude that $\lambda_{max}(\frac{J^{\frac{1}{2}}AJ^{-\frac{1}{2}}+J^{-\frac{1}{2}}A^{⊤}J^{\frac{1}{2}}}{2})<0$, that is, $x^{⊤}(J^{\frac{1}{2}}AJ^{-\frac{1}{2}}+J^{-\frac{1}{2}}A^{⊤}J^{\frac{1}{2}})x<0$ holds for each $x\in R^{n}$, which is equivalent to ${\left(J^{-\frac{1}{2}}x\right)}^{⊤}(JA+A^{⊤}J)(J^{-\frac{1}{2}}x)<0$, that is, we need $JA+A^{⊤}J<0$. And it is the same with *B*, $JB+B^{⊤}J<0$. With all the conditions above, we need to verify the existence and uniqueness of the system's solution. We have

$$\lim_{z^{⊤}x\to 0}z^{⊤}[(A-B)x+(a-b)]=\lim_{k\to 0}z^{⊤}[h+k(A-B)z+(a-b)]=\lim_{k\to 0}kz^{⊤}(A-B)z-\gamma z^{⊤}J^{-1}z,$$

which is smaller than zero, and guarantees that (17) has a unique solution. □

## Footnotes

1 We highlight that σ(⋅) can be also continuous, non-decreasing, and piecewise differentiable. The arguments also hold. In this case, the gradient operator should be changed to Clark generalized gradient. Readers can refer to Part A in Sec. 2 in [28] and [24] for details.
